# Determinants of habitat suitability models transferability across geographically disjunct populations: Insights from *Vipera ursinii urs* *inii*

**DOI:** 10.1002/ece3.7294

**Published:** 2021-03-17

**Authors:** Francesco Cerasoli, Aurélien Besnard, Marc‐Antoine Marchand, Paola D'Alessandro, Mattia Iannella, Maurizio Biondi

**Affiliations:** ^1^ Department of Life, Health and Environmental Sciences—Environmental Sciences Sect. University of L'Aquila L'Aquila Italy; ^2^ CEFE UMR 5175 CNRS PSL Research University Université Paul‐Valéry Montpellier, EPHE Montpellier France; ^3^ Conservatoire d'Espaces Naturels de Provence‐Alpes‐Côte d'Azur Pôle Alpes du Sud Sisteron France

**Keywords:** extrapolation, habitat suitability models, niche differentiation, spatial thinning, transferability, *Vipera ursinii ursinii*

## Abstract

Transferability of habitat suitability models (HSMs), essential to accurately predict outside calibration conditions, has been seldom investigated at intraspecific level. We targeted *Vipera ursinii ursinii*, a meadow viper from southeastern France and central Italy, to assess determinants of transferability among geographically disjunct populations. We fitted HSMs upon occurrences of the Italian and French populations separately, as well as on the combined occurrence dataset. Internal transferability of HSMs, on spatially independent test data drawn from the calibration region, and their external transferability on the geographically disjunct populations were evaluated according to (a) use of full or spatially rarefied presence datasets; (b) ecology‐driven or statistics‐driven filtering of predictors; (c) modeling algorithm, testing generalized additive models and gradient boosting models; and (d) multivariate environmental novelty within test data. Niche overlap between French and Italian populations was also tested. Niche overlap was low, but niche divergence between the two populations’ clusters was not corroborated. Nonetheless, wider niche breadth and heterogeneity of background environmental conditions characterizing the French populations led to low intercluster transferability. Although models fitted on the combined datasets did not attain consistently higher internal transferability than those separately fitted for the French and Italian populations, ensemble projection from the HSMs fitted on the joint occurrences produced more consistent suitability predictions across the full range of *V. u. ursinii*. Spatial thinning of occurrences ameliorated internal transferability but did not affect external transferability. The two approaches to predictors filtering did not differ in transferability of the respective HSMs but led to discrepant estimated environment–occurrence relationships and spatial predictions, while the two algorithms attained different relative rankings depending on the considered prediction task. Multivariate novelty of projection sites was negatively correlated to both internal transferability and external transferability. Our findings clarify issues researchers should keep in mind when using HSMs to get predictions across geographically disjunct populations.

## Graphical abstract

Transferability of habitat suitability models (HSMs) has been a largely debated topic in predictive ecology for the last two decades. We investigated the factors affecting HSMs https://CRAN.R-project.org/package=ncf transferability across geographically disjunct populations using as a model system *Vipera ursinii ursinii*, a meadow viper from southeastern France and central Italy. Spatial thinning of occurrences improves internal transferability, while incomplete coverage of the taxon realized niche within calibration data hampers external transferability in case of multivariate novelty in test data. Algorithm chosen to fit the HSMs and the approach used to filter candidate predictors should be also taken into account to comprehensively interpret model predictions.

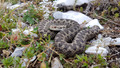

## 1 INTRODUCTION

The distribution of species is shaped by the interplay across space and time of abiotic and biotic factors. The presence of a species in a certain region requires the realized environment (i.e., the combination of abiotic conditions characterizing a geographical area) matching its physiological tolerances (i.e., its fundamental niche) (Guisan et al., 2014). The lack of physical barriers impeding the species to reach favorable habitats and the availability of trophic resources represent additional critical factors for the persistence of populations (Soberón & Nakamura, 2009). Usually, not all the areas matching such requirements are occupied. For instance, species which are nowadays patchily distributed may have continuously occurred in the past across wide extents: dramatic climate change, such as during the Quaternary glaciations, may have driven environmental conditions within portions of their historic range out of their fundamental niche, leading to local extinctions (Hewitt, 2000; Iannella et al., 2017). When existing clusters of populations are geographically segregated, with scant possibility of gene flow, intraspecific diversification may arise due to genetic drift. Moreover, different abiotic conditions characterizing the disjunct occurrence areas, though still comprised within the species’ fundamental niche, and compositional differences of the respective biotic communities may boost intercluster diversification (Borer et al., 2011; Maia‐Carvalho et al., 2018; Serra‐Varela et al., 2015). The degree to which disjunct distribution of intraspecific lineages or closely related species is associated with divergence or conservatism of the respective environmental niches has been widely studied in recent years (Martínez‐Freiría et al., 2020; McCormack et al., 2010; Rato et al., 2015).

Habitat suitability models (HSMs) are correlative models that estimate the relationship between the target biological entity and environmental predictors (e.g., climate, vegetation, topography, trophic resources) based on information about occurrence patterns of such entity across the study area (Guisan et al., 2017). HSMs, also termed species distribution models (SDMs) or ecological niche models (ENMs) depending on whether the “modeling landscape” is a concrete geographical space or only an environmental one (Owens et al., 2013), are increasingly used to investigate the way historical and current abiotic and biotic drivers shape distributions at different scales (Acevedo et al., 2012; Iannella et al., 2018; Reino et al., 2017). Indeed, once the entity–environment relationship has been estimated upon the available occurrence data (i.e., model calibration), HSMs may be projected to new scenarios (i.e., model transfer), such as past or future temporal horizons or distant geographical regions, predicting how suitable conditions to the entity would be distributed therein (Elith & Leathwick, 2009).

Given this predictive potential of HSMs, various researches aimed at evaluating their spatial and/or temporal transferability (Heikkinen et al., 2012; Qiao et al., 2019; Veloz et al., 2012), namely the degree to which their predictions would be reliable and biologically realistic outside calibration conditions. Transferability of HSMs has been related to several factors, such as sampling bias in calibration data, species’ traits, model complexity, and similarity of environmental conditions between calibration and projection spatiotemporal scenarios (Werkowska et al., 2017; Yates et al., 2018). Particularly, when environmental conditions in the projection region/timeframe fall outside the range of values characterizing one or more variables within calibration data (i.e., univariate/strict novelty), or include novel combinations of values which do not exceed the calibration range considering each variable separately (i.e., multivariate novelty), HSMs are forced to extrapolate the estimated entity–environment relationship into these “unknown territories,” exacerbating the risk of spurious predictions (Zurell et al., 2012).

Some populations’ clusters of rare or cryptic species showing patchy distributions may be more extensively investigated than others, as emerged for some European bats (Rebelo et al., 2010), viperids (Mizsei et al., 2016), beetles (Bosso et al., 2013), and plants (Fois et al., 2015). In this context, calibrating HSMs on a thoroughly sampled portion of the species’ range and projecting them to less explored regions seems a promising strategy to guide new field campaigns in these latter (Fois et al., 2018), possibly leading to the discovery of previously unknown populations (Mizsei et al., 2016; Rebelo & Jones, 2010). However, if calibration data do not fully cover the range of environmental conditions characterizing the species’ realized niche (i.e., its fundamental niche constrained by realized environment and biotic interactions), HSMs may fail in predicting species’ potential presence in areas actually suitable but not surveyed yet (Guisan et al., 2017; Thuiller et al., 2004). Moreover, the possibility of noticeable differences in the realized environment between calibration and projection areas usually increases with their geographic distance, worsening projection pitfalls due to extrapolation requirements (Qiao et al., 2019). Finally, if populations’ clusters have been segregated for dozens of millennia, possible intraspecific niche differentiation may have occurred in the meanwhile, making the HSMs calibrated on a cluster difficultly transferable to a different one (Carretero & Sillero, 2016).

HSMs have been increasingly applied at intraspecific level, for instance to quantify the contribution of niche divergence to the genetic diversification of population lineages with allopatric distribution (Maia‐Carvalho et al., 2018; Martínez‐Freiría et al., 2020) or to assess the relative weight of abiotic and biotic drivers in microhabitat selection (Peñalver‐Alcázar et al., 2016). Nonetheless, to the best of our knowledge, the factors affecting the transferability of HSMs among populations’ clusters having been geographically segregated for a long time were seldom targeted, although some previous attempts to unveil them do exist (Acevedo et al., 2014; Carretero & Sillero, 2016).

To fill in this gap of knowledge, we investigated HSMs transferability among geographically segregated populations targeting *Vipera ursinii ursinii* (Bonaparte, 1835) as a model system. Phylogeography of *V. ursinii* was shaped by the Quaternary glacial–interglacial cycles, with Balkan Peninsula probably acting as diversification center (Ferchaud et al., 2012): Since 2.1 m.y.a., the ancestral lineage leading to the current Italian and French *V. u. ursinii* and Croatian *V. u. macrops* (Méhely, 1911) populations moved northwards from Greece to Croatia and Slovenia, while the lineage from which *V. u. moldavica* Nilson, Andrén and Joger, 1993 and *V. u. rakosiensis* Méhely, 1893 diversified emerged between Greece and Montenegro and later moved to the northeast toward the steppes of Hungary, Romania, and Moldova (Ferchaud et al., 2012; Zinenko et al., 2015). Successively, *V. u. ursinii* colonized the Alpine arc and then spread southwards in Italy (Ferchaud et al., 2012). The current disjunct distribution of *V. u. ursinii* between western Alps in southeastern France and the Apennines massif in central Italy, with populations occurring in isolated patches dominated by alpine and subalpine grasslands (Luiselli, 2004; Lyet et al., 2013), is assumed to derive from contractions of suitable habitats during the Pleistocene interglacial periods, when forests replaced steppes at lower altitudes (Ferchaud et al., 2012). Since the divergence between the Italian and French *V. u. ursinii* populations started around 0.6 m.y.a. (Ferchaud et al., 2012), niche differentiation between them might have occurred: In this case, HSMs fitted on occurrences from the French populations would likely be poorly transferable to the Italian ones, and vice versa.

Therefore, because of its distributional patterns and phylogeographic history, we chose *V. u. ursinii* to investigate the factors affecting transferability among disjunct populations. For this purpose, we built state‐of‐the‐art HSMs upon occurrence data of the French populations, of the Italian ones, and of the two populations’ clusters together, using either all the available occurrences or spatially rarefied subsets thereof. Moreover, since the way predictors are chosen is deemed to be an important driver of predictive performance (Bucklin et al., 2015; Werkowska et al., 2017), HSMs were fitted using either a set of uncorrelated predictors entirely filtered through statistical procedures or a set of predictors resulting from a preliminary check for multicollinearity followed by critical selection of variables linked to *V. u. ursinii* known autoecology. Once HSMs were calibrated, internal transferability (i.e., predictive performance on test data drawn from the calibration region) was evaluated on spatially independent test samples, while external transferability (i.e., predictive performance on a disjunct geographic area) was assessed validating the HSMs built for the French populations on the Italian ones and vice versa. Moreover, we implemented the analytical framework proposed by Broennimann et al. (2012) to investigate whether *V. u. ursinii* disjunct distribution is associated with some degree of divergence between the environmental niches of the French and Italian populations.

## 2 METHODS

### 2.1 Occurrence data, spatial thinning, and pseudoabsences

**FIGURE 1:**
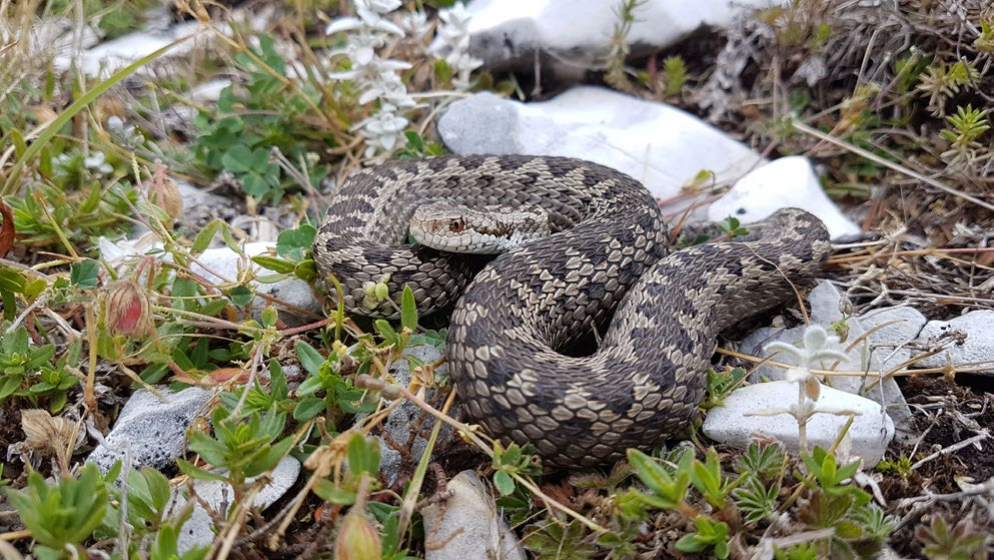
A specimen of *Vipera ursinii ursinii* (Bonaparte, 1835) pictured by one of the authors on emerging rocks within a mountainous meadow in central Italy

We gathered *V. u. ursinii* (Figure 1) occurrence data (GPS coordinates or specific toponym for which uncertainty about observation point was less than 1 km^2^) ranging from 1980 to 2017. Presence records for the Italian populations were extracted from the database on European occurrences of *V. ursinii* built by Console et al. (2020); for the French populations, we integrated occurrences from this database with observations extracted from the “SILENE faune” platform managed by the CEN‐PACA (Conservatoire d’Espaces Naturels de Provence‐Alpes‐Côte d'Azur). This way, we collected 1,376 presence records for the French populations and 302 records for the Italian ones. Based on these two sets of occurrences, we used the “alphahull” (Rodríguez Casal & Pateiro López, 2010) R (R Core Team, 2019) package to draw alphahull‐based polygons, which provide less bias‐prone range estimates than minimum convex polygons (Burgman & Fox, 2003): Such polygons were successively overlaid on maps obtained from HSMs projection across the study area (see “Ensemble predictions and climate–occurrence relationships”) to more intuitively show how predicted suitability was distributed within the estimated range of the two populations’ clusters.

Occurrence records were then allocated to three different calibration groups: (a) “France,” containing only occurrences of the French populations; (b) “Italy,” containing only the Italian records; (c) “Joint,” comprising occurrences from both populations’ clusters.

Occurrences were spatially rarefied through the “spThin” R package (Aiello‐Lammens et al., 2015): We performed three thinning replicates for each populations’ cluster (i.e., “France” and “Italy”), setting 1.5 km as minimum allowed distance between two thinned occurrences to rarefy presence points falling within neighboring raster cells of the predictors (see “Environmental predictors”); then, for each cluster, we selected the replicate providing the highest number of residual presence points, to preserve as much information as possible about the environment‐occurrence relationship. The thinned occurrences retained for the French and Italian populations were then joined to build the “Joint” thinned dataset.

Thus, we used six different presence datasets, resulting from three calibration groups * two occurrence data sizes (hereafter, “Full” and “Thin”), to calibrate the HSMs.

Since the algorithms used to fit the HSMs (see “Model fitting and evaluation”) require both presences and absences, we generated, for each presence dataset, geographically buffered pseudoabsences following the “1° far” approach, claimed to assure good performances with the chosen algorithms (Barbet‐Massin et al., 2012). By means of the “rgdal” R package (Bivand et al., 2019), we first drew an inner buffer polygon of 1.5 km radius and an outer buffer polygon of 120 km (approximately 1°) radius around each occurrence; the two polygons were subsequently subtracted to get a final buffer ranging from 1.5 to 120 km around each occurrence; the so‐obtained buffer polygons were merged and then clipped to the boundaries of the study area, to finally generate 10 sets of 1,000 pseudoabsences each by random selection within the merged and clipped polygon. This way, neither a presence point could be incidentally selected as pseudoabsence, nor pseudoabsences could fall too close to any occurrence site. We finally joined each presence dataset with the corresponding 10 sets of pseudoabsences, obtaining the presence–pseudoabsence (hereafter Pres‐PseudoAbs) datasets used for model fitting.

**FIGURE 2:**
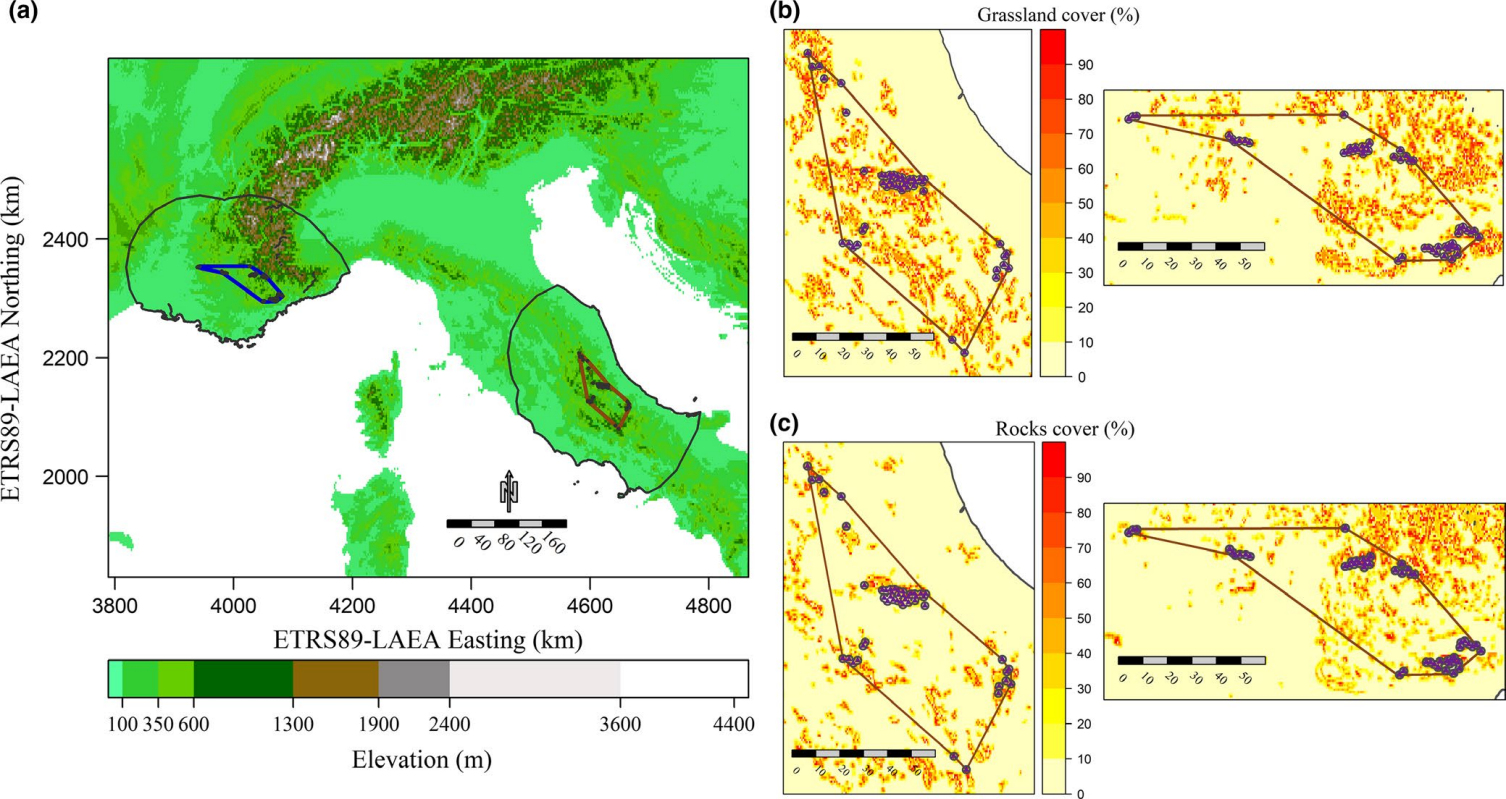
(a) Altitudinal gradient characterizing the study area; alphahull‐based polygons estimating the current range of the Italian (brown) and French (blue) *Vipera ursinii ursinii* populations are overlaid on the map, along with the background polygons (gray contour) from which the pseudoabsences needed to fit the HSMs were generated. (b) Raster maps representing the percent cover of grasslands within each cell in the occurrence range (and its surroundings) of the Italian (left) and French (right) populations. (c) Raster maps representing the percent cover of rocky or sparsely vegetated areas within each cell in the occurrence range (and its surroundings) of the Italian (left) and French (right) populations

Elevation gradient across the study area, alphahull‐based range estimates for the French and the Italian populations, and the respective buffer polygons from which pseudoabsences were drawn are show in Figure 2a.

### 2.2 Environmental predictors

#### 2.2.1 Climate‐related predictors

Climatic variables represent important predictors of the environmental suitability for herptiles (Guisan & Hofer, 2003; Lunghi et al., 2018; Mizsei et al., 2016). Raster files of nineteen temperature‐ and precipitation‐related variables (Bio1‐Bio19) at 30 arc‐seconds resolution (~1 km^2^ at the equator) were downloaded from WorldClim 2.0 online repository (Fick & Hijmans, 2017). From the same repository, we also downloaded raster files of monthly averaged daily solar radiation (kJ m^−2^ day^−1^) and wind speed (m/s) at the same resolution and processed them in R to obtain ten new variables related to (a) annual trends, represented by yearly averaged daily solar radiation (Srad_Ann_Mean) and wind speed (Wspeed_Ann_Mean) and their standard deviation (Srad_Ann_Sd and Wspeed_Ann_Sd); (b) activity period of *V. u. ursinii*, which goes from late spring to early autumn (Lisse et al., 2012; Luiselli, 2004), represented by mean and standard deviation of daily solar radiation and wind speed during the May–October period (Srad_MayOct_Mean, Srad_MayOct_Sd, Wspeed_MayOct_Mean, and Wspeed_MayOct_Sd); (c) solar radiation extremes, namely mean daily solar radiation of the least sunny month (Srad_LSM) and of the sunniest month (Srad_MSM). The raster files of all these climate‐related variables were then projected from the original WGS84 geographic coordinate system to ETRS89‐LAEA to maintain equal‐sized cells across the latitudinal range of the study area.

#### 2.2.2 Habitat‐related predictors

Previous studies implementing HSMs on *V. u. ursinii* (Lyet et al., 2013) and other *Vipera* species (Santos et al., 2006) included as predictors also vegetation types, land use, and human disturbances, which actually affect the composition of animal communities and the distribution of single taxa at regional‐to‐local scales (Iannella et al., 2016; Luoto et al., 2007). Here, to derive predictors representing habitat types linked to *V. u. ursinii* autoecology, we downloaded raster files of Corine Land Cover (“CLC”) data at 100‐m resolution from the Copernicus Land Monitoring Service website (https://land.copernicus.eu/pan‐european/corine‐land‐cover), for the years 1990, 2000, 2006, 2012, and 2018. First, we calculated in R the proportion of *V. u. ursinii* occurrence localities experiencing a shift in land cover classification among the five time slices: land cover changes involved only 11% of localities and mostly referred to 1990 CLC, indicating that no noticeable habitat modifications occurred within *V. u. ursinii* presence localities during the last 30 years; thus, considering also that 96.7% of the Italian occurrences and 98.3% of the French ones were recorded after 1990, we choose 2012 as reference year for land cover (hereafter, “CLC_2012”). Then, we computed the proportion of occurrence localities falling within each CLC_2012 class: Since almost 80% of presence records fall within “Natural grasslands,” “Bare rocks,” and “Sparsely vegetated areas,” we aggregated through bilinear interpolation the raster cells of CLC_2012 to the same resolution of climate‐related rasters and derived two habitat‐related predictors: (a) the percent cover of the “Natural grasslands” class within each aggregated raster cell (hereafter termed “Grasslands”) and (b) the percent cover of the “Bare rocks” and “Sparsely vegetated areas” classes within each aggregated cell (hereafter, “Rocks_SparseVeg”).

#### 2.2.3 Ecology‐based versus statistics‐based filtering of predictors

Joining climate‐related variables and habitat‐related ones, we considered a total of 31 candidate predictors. Since a high number of collinear variables may lead to unstable estimation of model parameters and biased contributions of the single predictors to the environment–occurrence relationship (Dormann et al., 2013), we first individuated the pairs of predictors showing Pearson's *r* correlation coefficient ≥ 0.7 across the study area extent.

Some authors suggest that selecting the variable presumed to be more significant for the species’ autoecology from each pair of correlated predictors makes HSMs predictions less bias‐prone and more easily interpretable (Rissler & Apodaca, 2007). Differently, others claim that hidden correlation structures may persist after a preliminary filtering of predictors based on pairwise correlations (Guisan et al., 2017) and suggest more statistically robust techniques such as the variance inflation factor (VIF) analysis to filter candidate predictors (Guisan et al., 2006; Werkowska et al., 2017). Here, to evaluate the effect of different predictor filtering approaches upon HSMs internal and external transferability, we generated two sets of predictors. The former, hereafter named “Ecol”, was built selecting the variables deemed to more directly influence *V. u. ursinii* ecological requirements among the ones showing multicollinearity issues (i.e., Pearson's *r* ≥ 0.7). The second, hereafter termed “VIF,” derived from a two‐stage procedure. First, we performed a VIF analysis across the study area and discarded the variables exceeding the recommended threshold of VIF = 10 (Guisan et al., 2017). Then, since multicollinearity issues could raise even once dropped the variables being highly correlated at the study area scale (Cerasoli et al., 2019; Guisan et al., 2017), we extracted the values of the initially retained variables in correspondence of the points comprised in the Pres‐PseudoAbs datasets generated for each combination of calibration group * occurrence data size and performed new VIF analyses: The variables not exceeding VIF = 10 for all the combinations were finally selected as input predictors.

### 2.3 Model fitting and evaluation

We selected generalized additive models (GAMs), a regression‐based approach frequently used in ecological modeling (Guisan et al., 2002), and gradient boosting models (GBMs), also known as boosted regression trees (BRTs) (Elith et al., 2008), to fit the HSMs. GAM is based on smoothing functions permitting to fit the modeled response to the data when their relationship would be difficultly fitted through standard parametric methods (Guisan et al., 2017). Differently, GBM implements stochastic boosting to build several simple regression trees in a forward stagewise fashion, focusing at each iteration on residuals from the set of trees built up to that point, finally combining them in a single optimized model (Elith et al., 2008).

We chose GAM and GBM because these algorithms attained high predictive performance in previous transferability‐focused studies considering multiple species (Heikkinen et al., 2012) or virtual species (Qiao et al., 2019), but to the best of our knowledge, their transferability at intraspecific level among disjunct populations has not been evaluated yet, contrarily to some presence‐only algorithms (Carretero & Sillero, 2016).

Here, GAMs were fitted by means of the “gam” R package (Hastie, 2018), setting binomial distribution and cubic‐spline smoother with maximum smoothing degree (“s”) of 3; the initial model was then refined by means of stepwise selection (with both the forward and backward directions tested) of predictors via Akaike's information criterion (AIC) (Guisan et al., 2017). GBMs were instead fitted through the “gbm” R package (Greenwell et al., 2019), setting learning rate = 0.001, three‐way interactions (i.e., “tree complexity” = 3), maximum number of trees = 10,000, and fivefold internal cross‐validation.

Occurrence data often show internal dependence structures possibly leading to model overfitting, incorrect estimation of errors, and inflated performance metrics (Roberts et al., 2017; Veloz, 2009). In our case, the restricted range of the two populations’ clusters could exacerbate spatial autocorrelation issues; in such situations, the commonly used cross‐validation approaches (e.g., k‐fold or repeated split‐sample) would lead to overoptimistic error estimates and differences in predictive performance between nearby and distant test localities (Roberts et al., 2017; Valavi et al., 2019). Thus, we opted for spatial blocking cross‐validation. To define an appropriate block size we fitted a GAM and a GBM model for each Pres‐PseudoAbs dataset, we computed residuals from the predictions of these “full data” HSMs on calibration data, and we subsequently drew correlograms on residuals through the “ncf” R package (Bjornstad, 2019) to visualize the variations of Moran's Index at increasing interpoint distances (10 km pace): The distance at which Moran's Index stably approaches 0 represents the range of spatial autocorrelation, and block size should be greater than this distance to adequately assess prediction errors (Roberts et al., 2017; Valavi et al., 2019). Once individuated the optimal size, blocks were built through the “blockCV” R package (Valavi et al., 2019) and assigned to the training and test folds in a checkerboard fashion: this blocking structure makes environmental heterogeneity more evenly distributed between the training and the test fold compared with the grouping of contiguous blocks into a same fold, which would favor extrapolation‐related low predictive performance on test data (Roberts et al., 2017).

Our modeling framework resulted in a total of 480 HSMs (6 presence datasets * 10 pseudoabsence replicates * 2 sets of predictors * 2 algorithms * 2 blocks‐to‐folds assignments).

We assessed internal transferability of the obtained HSMs on the respective test folds (hereafter, “SpBlock CV”) by means of two metrics, the area under the curve (AUC) of the receiver operating characteristic (ROC) plot (Fielding & Bell, 1997) and the Continuous Boyce Index (Hirzel et al., 2006). AUC, a discrimination metric widely used in HSM literature (Cerasoli et al., 2017; Mammola et al., 2018; Yates et al., 2010), is measured plotting true‐positive rate (i.e., sensitivity) against false‐positive rate (i.e., 1‐specificity) along increasing suitability thresholds. When HSMs are built using pseudoabsences instead of true absences, AUC can only provide comparative evaluations among HSMs built for a same entity through various algorithms and/or parameterizations, or among HSMs built for entities whose occurrences and pseudoabsences are collected from a same extent (Jiménez‐Valverde et al., 2013; Lobo et al., 2008). Differently, the Continuous Boyce Index considers how occurrences are distributed within the range of predicted suitability: A window of fixed width is iteratively moved along the suitability range, each time computing the predicted‐to‐expected ratio *Fi* (i.e., proportion of occurrences divided by the proportion of raster cells falling within the window) and plotting it against the mean/median suitability value of the window; the Spearman correlation coefficient between *Fi* and the mean/median suitability values is then calculated (Hirzel et al., 2006). The Continuous Boyce Index plot and the respective correlation coefficient (hereafter indicated as *B*), ranging between −1 (counter‐predictions) and 1 (optimal predictions), can be used to contrast predictive performance of HSMs calibrated on Pres‐PseudoAbs data from different areas, as this metric refers only to presences (Guisan et al., 2017). Therefore, we used AUC to assess accuracy differences between HSMs fitted through GAM and GBM, and between HSMs fitted upon the two different predictors sets, for a same calibration group * occurrence data size combination, while we focused on Continuous Boyce Index to evaluate predictive performances across calibration groups and between “Full” and “Thin” presence datasets. For each of the 480 HSMs, we computed AUC on test fold through the “PresenceAbsence” R package (Freeman & Moisen, 2008), whereas we computed *B* on test fold by means of the “ecospat” R package (Broennimann et al., 2018).

To analyze external transferability between the French and the Italian populations (hereafter, “External validation”), we projected each of the spatially blocked HSMs fitted for a populations’ cluster on a randomly chosen Pres‐PseudoAbs dataset of the other, successively computing AUC and *B*.

Possibly emerging differences in predictive performance between algorithms, sets of predictors, size of occurrence datasets, calibration groups, and validation methods (i.e., “SpBlock CV” vs. “External validation”) were statistically tested at *α* = 0.05. In case normality of residuals from a linear model relating the relevant performance metric (i.e., AUC or *B*) to the target factors was not clearly rejected by the Shapiro–Wilk test and inspection of Q–Q plots, we verified whether the other conditions for parametric tests (e.g., homoscedasticity) were met as well; otherwise, we opted for nonparametric tests such as the Kruskal–Wallis test and subsequent Wilcoxon signed‐rank test with Benjamini–Hochberg correction when more than two classes had to be compared, or the Mann–Whitney *U* test for two‐classes factors.

Since extrapolation has been advocated as a primary issue for transferability (Yates et al., 2018), we assessed multivariate novelty that the HSMs faced when projected on test data: For this purpose, we used environmental overlap masks (Zurell et al., 2012) permitting to identify the projection sites showing novel combinations of values of the predictors compared with the calibration sites (i.e., nonanalog sites). Indeed, while most of previous transferability‐focused researches mainly highlighted the issues raising from univariate novelty (e.g., Owens et al., 2013), we aimed to evaluate whether the less apparent multivariate novelty could be a strong correlate of troubling model transfer as well. Thus, we checked for possible significant correlation between HSMs internal and external transferability (considering both AUC and *B*) and the degree of extrapolation (expressed as percentage of nonanalog test sites) through correlation *t* tests. Rank‐based inverse normal transformation was applied to AUC, *B,* and percentage of nonanalog test sites to approach normality before running correlation *t* tests.

### 2.4 Ensemble predictions and climate–occurrence relationships

Previous researches suggested that predictions based on ensemble of HSMs could be more informative than predictions from single models (Araújo & New, 2007; Marmion et al., 2009), though Hao et al. (2020) showed that ensemble predictions may not outperform those obtained from properly tuned single HSMs. Here, we generated ensemble predictions across the study area for each combination of calibration group * occurrence data size * set of predictors through a weighted average of predictions (Weighted_Avg_HS) from the corresponding HSMs, based on their predictive performance (Marmion et al., 2009): HSMs showing *B* ≥ 0.7 within “SpBlock CV” were selected and their predictions weighted upon the attained *B* value. Moreover, since variability in suitability patterns predicted by the component HSMs is informative for a comprehensive evaluation of ensemble predictions (Araújo & New, 2007; Guisan et al., 2017), we also computed weighted standard deviation of suitability (Weighted_StdDev_HS) values across the study area from the HSMs selected for ensemble predictions, through the formula:

(1) $$\text{Weighted}\_\text{StdDev}\_\text{HS}=\sqrt{\frac{\sum_{i=1}^{N}B_{i}*{\left({\text{HS}}_{i}‐{\text{HS}}_{\text{WA}}\right)}^{2}}{\frac{N‐1}{N}*\sum_{i=1}^{N}B_{i}}}$$

where *N* is the number of HSMs selected for ensemble predictions, *Bi* represents the Continuous Boyce Index obtained by the *i‐*th HSM within “SpBlock CV,” HS*_i_* is the suitability value predicted by the *i‐*th HSM for a given site, and HS_WA_ is the weighted average suitability value obtained from the *N* HSMs for the same given site.

For each combination of calibration group * occurrence data size * set of predictors, importance scores of variables were extracted from the HSMs selected for ensemble predictions through the algorithm‐independent procedure implemented in the “biomod2” (Thuiller et al., 2019) R package. Raw scores were normalized to percent contributions and weighted based on the *B* value attained by the corresponding HSM within “SpBlock CV,” obtaining weighted average contributions (Weighted_Avg_Imp); weighted standard deviation of importance scores (Weighted_StdDev_Imp) was also computed through a formula analogous to Equation 1.

To explore variability in the predictor–suitability relationships across the combinations of calibration group * occurrence data size * set of predictors, we individuated for each combination the HSM attaining the highest *B* value within “SpBlock CV”; from this latter, we drew inflated response curves (Zurell et al., 2012) of the three predictors showing the lowest coefficient of variation (i.e., coeff. var. = $\frac{\text{Weighted}\_\text{StdDev}\_\text{Imp}}{\text{Weighted}\_\text{Avg}\_\text{Imp}}$) among the five ones attaining the highest Weighted_Avg_Imp scores. Inflated response curves show how suitability changes along the range of each predictor in correspondence of different combinations of values (e.g., mean, minimum, 1st quartile) of the other predictors.

### 2.5 Niche overlap

We implemented the PCA‐Env approach described in Broennimann et al. (2012) using the “ecospat” package: a PCA performed on sites randomly sampled from the entire study area permits to derive a 2D space whose axes represent the components best summarizing environmental variability across the study area; within the gridded 2D environmental space, the “niche occupancy” of the target entity is estimated comparing the kernel‐smoothed density of occurrence of the entity in each cell with the density of available environmental conditions within the same cell. We extracted kernel‐smoothed niche occupancies for the Italian and French populations, subsequently computing Schoener's *D* metric (Schoener, 1970) to estimate their overlap. Then, we performed the niche equivalency and niche similarity tests (Warren et al., 2008). The equivalency test compares the observed *D* to a set of *D* values from virtual niches obtained through random reallocation of occurrences between the two entities, and niche equivalency cannot be rejected unless the observed *D* falls outside the 95% of the simulated *D* values. Differently, within the similarity test simulated *D* values are computed comparing the actual niche of one entity to virtual niches estimated upon randomly selected points from the background environment available to the other entity (Di Cola et al., 2017): niches of the two entities are considered more or less similar than expected, given the respective realized environments, depending on whether the observed *D* is higher than the 95th percentile or lower than the 5th percentile of the simulated distribution.

For both tests, 1,000 virtual niches were created; for the similarity test, we considered as background area for each populations’ cluster the buffer polygon drawn around the respective “Full” occurrence dataset to generate pseudoabsences.

## 3 RESULTS

The spatial thinning led to the retention of 51 occurrences for the French populations and 49 occurrences for the Italian ones. Thus, while the “Full” datasets comprised 1,376 presence points for the “France” calibration group, 302 for the “Italy” group, and 1,678 for the “Joint” one, the corresponding “Thin” datasets comprised 51, 49 and 100 occurrences, respectively.

The two approaches to predictors filtering produced final sets differing both in the number of predictors and in the climatic trends represented (Table 1). Out of the nineteen temperature‐ and precipitation‐related variables (Bio1‐19), six were selected for the “Ecol” set while five were retained in the “VIF” one, with the two sets sharing only Bio9 (mean temperature of driest quarter) and Bio15 (precipitation seasonality). The variables related to solar radiation and wind speed during *V. u. ursinii* activity period (i.e., mean and standard deviation in the May–October timeframe) were chosen for the “Ecol” set once their collinearity with annual trends emerged; annual standard deviation of wind speed (Wspeed_Ann_Sd) was included as well because it was not correlated to the other predictors and variations in wind speed have been shown to affect herptiles’ occurrence patterns (Ortega et al., 2017; Sun et al., 2001). The two‐stage VIF‐based filtering approach, instead, led to the retention of annual mean and standard deviation of daily solar radiation (Srad_Ann_Mean and Srad_Ann_Sd), while wind speed was represented only by its standard deviation during *V. u. ursinii* activity period (Wspeed_MayOct_Sd). Percent cover of grasslands (“Grasslands”) and bare rocks or sparsely vegetated areas (“Rocks_SparseVeg”) is noticeably high (50%–90%) in most of *V. u. ursinii* French and Italian occurrence localities (Figure 2b,c), and these habitat‐related variables were retained both in the “Ecol” and in the “VIF” sets.

**TABLE 1:** Predictors selected to fit the HSMs following the two tested filtering approaches

Filtering of predictors	
Ecol	VIF
Bio2	Bio3
Bio9	Bio4
Bio10	Bio9
Bio15	Bio13
Bio16	Bio15
Bio18	Srad_Ann_Mean
Srad_MayOct_Mean	Srad_Ann_Sd
Srad_MayOct_Sd	Wspeed_MayOct_Sd
Wspeed_Ann_Sd	Grasslands
Wspeed_MayOct_Mean	Rocks_SparseVeg
Wspeed_MayOct_Sd	/
Grasslands	
Rocks_SparseVeg	

Note

“Ecol” indicates the set of predictors resulting from a preliminary check for pairwise collinearity through Pearson's *r* correlation coefficient, followed by critical evaluation of which variables could be more directly linked to the known autoecology of *Vipera ursinii ursinii*. “VIF” indicates the set of predictors obtained through a filtering procedure totally based on variance inflation factor analysis.

Correlograms showed that Moran's Index approached 0 within the 50–60 km distance bin for all the calibration group * occurrence data size combinations except the “Joint‐Full” one; for this latter, spatial autocorrelation became negligible (i.e., Moran's Index = 0 ± 0.05) only at 250–300 km interpoint distance (Figure S1); thus, we performed checkerboard blocking choosing 275 km as block size for the “Joint‐Full” combination, and 60 km as block size for the others (Figure S2).

Predictive performance in terms of AUC and Continuous Boyce Index (*B*) of the 480 fitted HSMs is reported, for both “SpBlock CV” and “External validation,” in a table provided as Supplementary Information.

Neither normality nor homoscedasticity was clearly fulfilled within any of the linear models relating the various considered factors to AUC and *B* values within “SpBlock CV” and “External validation” (see below), so we used nonparametric testing for subsequent comparisons.

**FIGURE 3:**
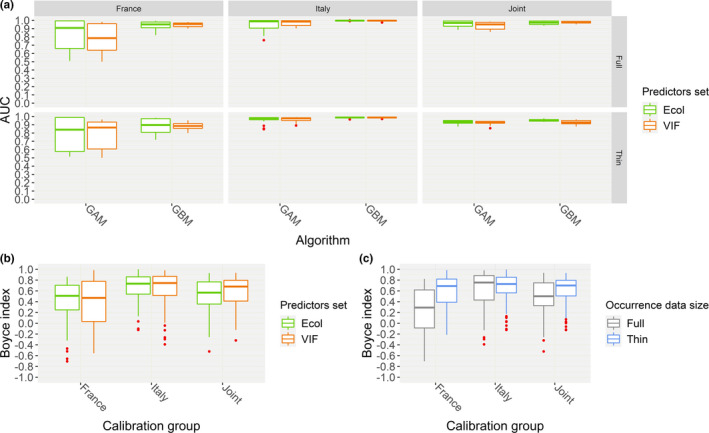
(a) Box and whiskers plots showing, for each combination of calibration group (“France,” “Italy,” “Joint”) * occurrence data size (“Full,” “Thin”), the AUC scores that the HSMs fitted through the GAM or GBM algorithm, using the “Ecol” or “VIF” set of predictors, attained when validated on spatially independent test samples drawn from the calibration region (“SpBlock CV”). (b) Box and whiskers plots showing the Continuous Boyce Index scores that the HSMs fitted upon the different calibration groups, using the “Ecol” or “VIF” set of predictors, attained in “SpBlock CV.” (c) Box and whiskers plots showing the Continuous Boyce Index scores that the HSMs fitted upon the different calibration groups, using the “Full” or “Thin” occurrence dataset, attained in “SpBlock CV”

No evident differences in AUC within “SpBlock CV” emerged between the HSMs fitted upon the “Ecol” set of predictors and those fitted upon the “VIF” one, while GBM apparently provided better discrimination than GAM (Figure 3a). The linear models AUC∼Algorithm∗Set of predictors fitted for each combination of calibration group * occurrence data size confirmed this trend, as only the algorithm factor emerged to significantly affect discrimination performance for most of the combinations (Table S1). Thus, one‐tailed Mann–Whitney *U* tests were performed to evaluate interalgorithm differences: GBM was confirmed to attain significantly higher AUC than GAM for “France‐Full” (*n* = 80, *U* = 1,022, *p* = 0.02), “Italy‐Full” (*n* = 80, *U* = 1,155.5, *p* < 0.001), “Italy‐Thin” (*n* = 80, *U* = 1,233.5, *p* < 0.001), and “Joint‐Thin” (*n* = 80, *U* = 1,094.5, *p* = 0.002), though estimated differences between the two algorithms were mild (est. diff. = 0.01–0.09).

The additive linear model B ∼ Calibration group + Occurrence data size + Algorithm + Set of predictors showed that, within “SpBlock CV”, *B* scores were not influenced by the set of predictors the HSMs were fitted upon (Figure 3b, Table S2). The refined linear model B ∼ Calibration group ∗ Occurrence data size + Algorithm confirmed significant effect of the other three factors on *B* scores; moreover, the positive effect of data thinning emerged as significantly less pronounced for the “Italy” and “Joint” groups than for the “France” one (Figure 3c, Table S2). The Kruskal–Wallis test confirmed significant differences across the three calibration groups (*χ^2^* = 38.6, *df* = 2, *p* < 0.001). The subsequent pairwise Wilcoxon signed‐rank tests showed that *B* from “Italy” group was significantly higher than *B* from the “France” (*p* < 0.001) and “Joint” (*p* < 0.001) ones, and that *B* from the “Joint” group was higher than *B* from the “France” one (*p* = 0.001).

One‐tailed Mann–Whitney *U* tests also confirmed that HSMs fitted upon the “Thin” datasets attained significantly higher *B* scores than those fitted upon the “Full” ones (*n* = 480, *U* = 33,297, *p* < 0.001, est. diff = 0.12), and that GBM obtained significantly higher *B* scores than GAM (*n* = 480, *U* = 37,190, *p* < 0.001, est. diff = 0.19).

**FIGURE 4:**
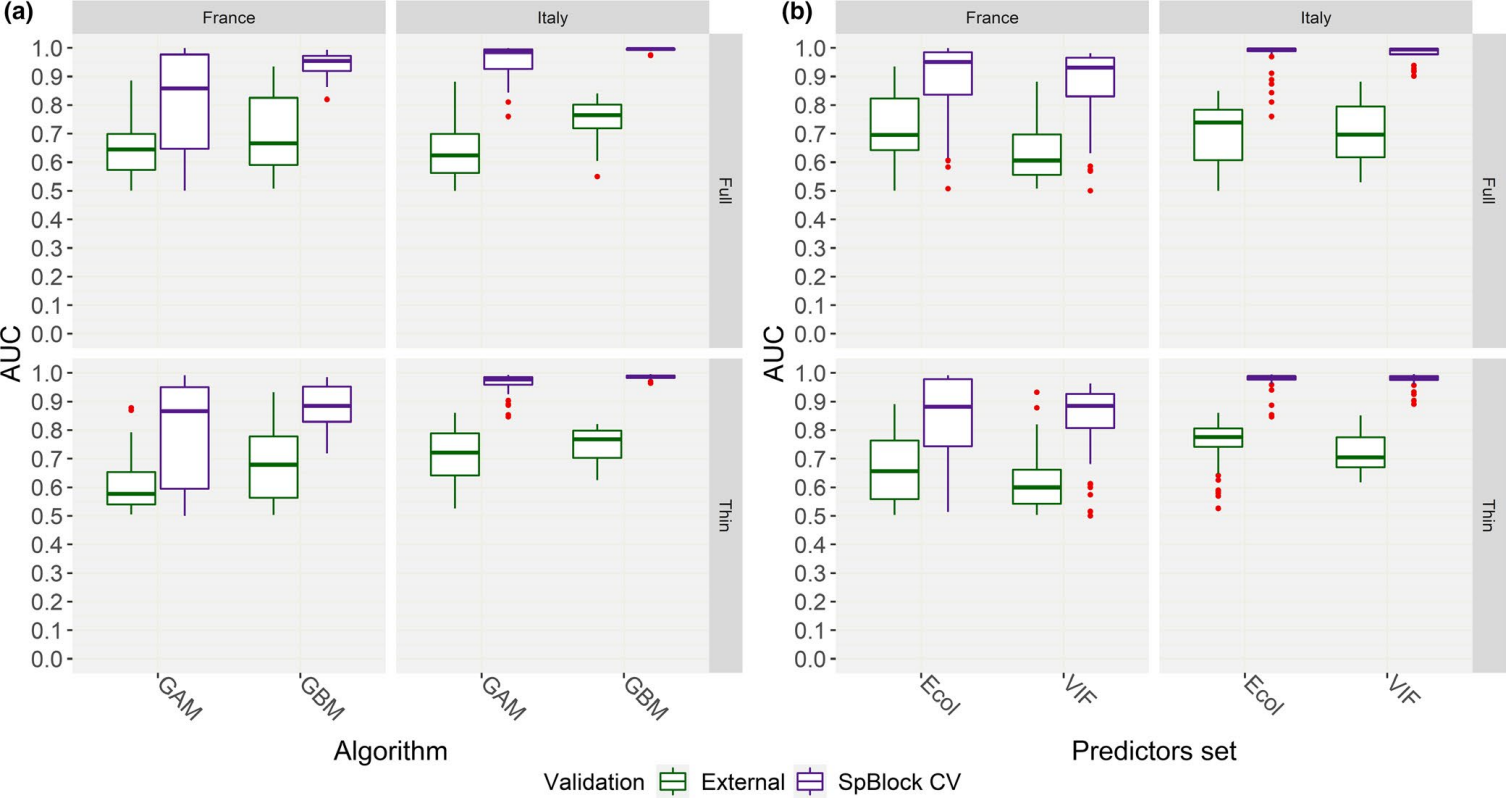
Box and whiskers plots showing, for each combination of calibration group (“France,” “Italy”) * occurrence data size (“Full,” “Thin”), the AUC scores that the HSMs attained when validated on spatially independent test samples drawn from the calibration region (“SpBlock CV”) or on test samples drawn from the geographically disjunct populations’ cluster (“External”), according to the (a) algorithm (GAM, GBM) and (b) set of predictors (“Ecol,” “VIF”) used to fit the model

Within “External validation,” the linear models AUC∼Algorithm∗Set of predictors showed that algorithm affected discrimination performance for all the calibration group * occurrence data size combinations (Figure 4a, Table S3), while the set of predictors emerged as influential only for “France‐Full” (AUC lower for “VIF” than for “Ecol”) and “Italy‐Full” (AUC higher for “VIF” than for “Ecol”) (Figure 4b, Table S3). One‐tailed Mann–Whitney *U* tests showed that GBM attained significantly higher AUC than GAM for all the combinations (“Italy‐Full”: *n* = 80, *U* = 1,230.5, *p* < 0.001; “France‐Full”: *n* = 80, *U* = 994.5, *p* = 0.03; “Italy‐Thin”: *n* = 80, *U* = 983, *p* = 0.04; “France‐Thin”: *n* = 80, *U* = 1,090.5, *p* = 0.003), with estimated differences ranging 0.03–0.13.

**FIGURE 5:**
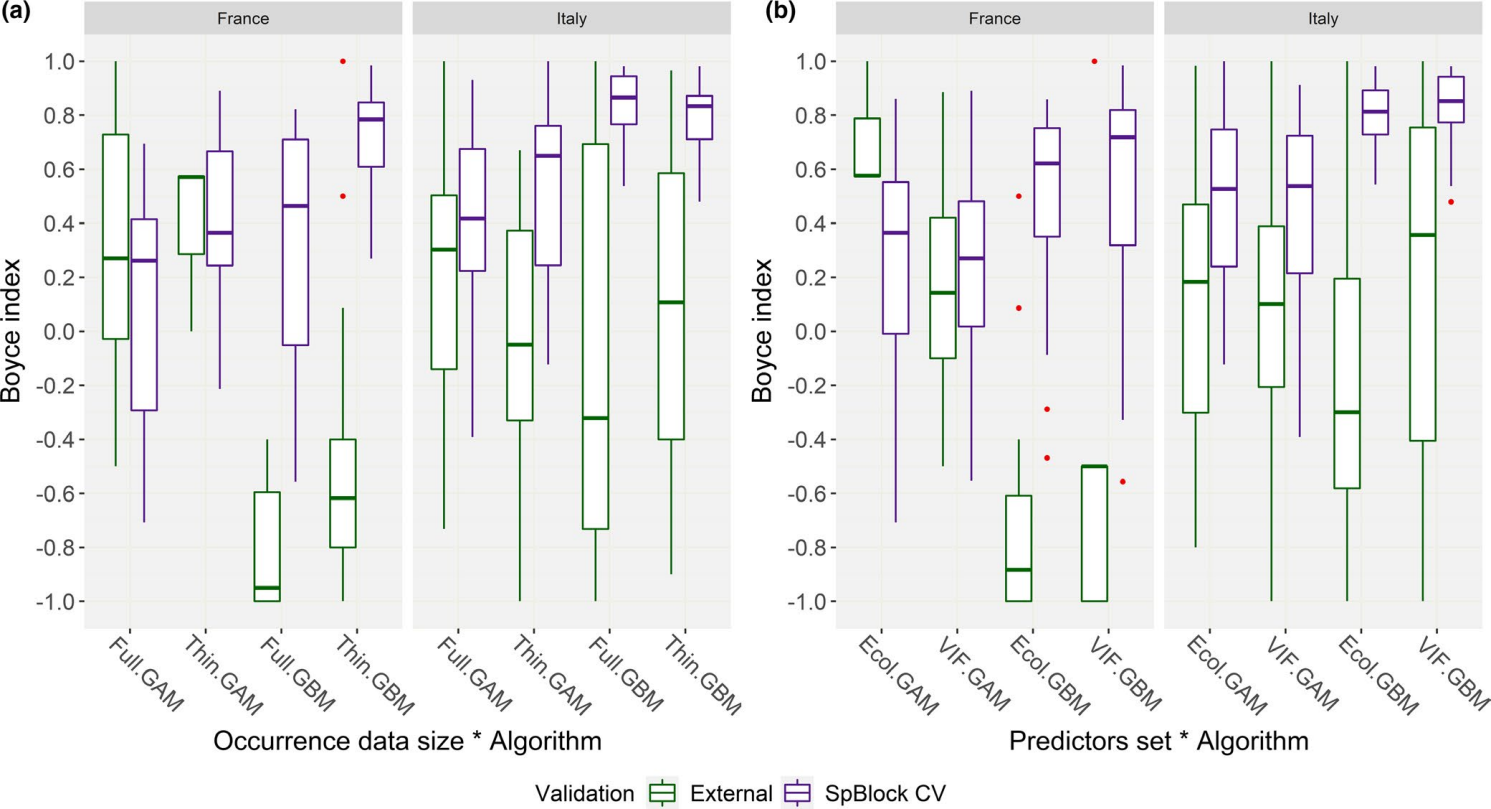
Box and whisker plots showing, for the “France” and “Italy” calibration groups, the Continuous Boyce Index scores that the HSMs attained when validated on spatially independent test samples drawn from the calibration region (“SpBlock CV”) or on test samples drawn from the geographically disjunct populations’ cluster (“External”), according to the (a) occurrence data size (“Full,” “Thin”) and (b) set of predictors (“Ecol,” “VIF”) used to fit the model

The additive linear model B ∼ Calibration group + Occurrence data size + Set of predictors + Algorithm fitted for “External validation” did not confirm the positive effect of spatial thinning on *B* scores which emerged within “SpBlock CV” (Figure 5a, Table S4). The refined linear model B ∼ Calibration group + Algorithm ∗ Set of predictors suggested that HSMs from the “Italy” group performed better than those from the “France” one in terms of *B*, while GBM appeared to perform worse than GAM; in this case, the chosen set of predictors did not emerge as influential factor, though marginally significant positive interaction resulted between GBM and “VIF” (Figure 5b, Table S4). One‐tailed Mann–Whitney *U* tests confirmed that HSMs fitted for the “Italy” group actually attained higher *B* scores than those fitted using only the French occurrences (*n* = 156, *U* = 1,909.5, *p* = 0.001, est. diff. = 0.43), and that GBM models obtained significantly lower *B* scores than GAM ones (*n* = 156, *U* = 3,030, *p* < 0.001, est. diff. = −0.35).

Comparing the two validation approaches, the HSMs fitted on the “Italy” and “France” groups attained higher predictive performance, in terms of both AUC and *B*, within “SpBlock CV” than within “External validation” (Figures 4, 5). The linear models AUC∼Validation approach (fitted for each calibration group * occurrence data size combination) and B∼Validation approach confirmed significant effect of validation approach on both metrics (Table S5). One‐tailed Mann–Whitney *U* tests showed that AUC was significantly higher in “SpBlock CV” than in “External validation” for all the combinations (“France‐Full”: *n* = 160, *U* = 5,402, *p* < 0.001, est. diff. = 0.25; “Italy‐Full”: *n* = 160, *U* = 6,348, *p* < 0.001, est. diff. = 0.26; “France‐Thin”: *n* = 160, *U* = 5,330, *p* < 0.001, est. diff. = 0.22; “Italy‐Thin”: *n* = 160, *U* = 6,397, *p* < 0.001, est. diff. = 0.22). Similarly, *B* values from “SpBlock CV” were significantly higher than those from “External validation” across the two populations’ clusters (*n* = 459, *U* = 36,700, *p* < 0.001, est. diff. = 0.62).

The percentage of test sites showing multivariate novelty was clearly higher within “External validation” (more than 99%) than within “SpBlock CV” (between 60% and 90%); contextually, variability in AUC values was higher in the former validation approach than in the latter, especially for the “France” group (Figure S3). Within “SpBlock CV,” the decrease in AUC and *B* scores at increasing multivariate novelty was more pronounced for GAM than for GBM (Figures S3, S4). While no differences emerged between “Ecol” and “VIF” considering predictive performance in terms of Continuous Boyce Index at increasing percentage of non‐analog test sites (Figure S4a), the lower multivariate novelty of the “Thin” datasets compared with the “Full” ones within “SpBlock CV” appeared to be associated with higher *B* scores for HSMs fitted on the thinned data (Figure S4b).

Once applied rank‐based inverse normal transformation of AUC, *B,* and percentage of nonanalog test sites, significant negative correlation between each of the two metrics and multivariate novelty (AUC: *r* = −0.61, *t* = −21.54, *df* = 798, *p* < 0.001; *B*: *r* = −0.43, *t* = −11.75, *df* = 617, *p* < 0.001) emerged across the two validation approaches.

**FIGURE 6:**
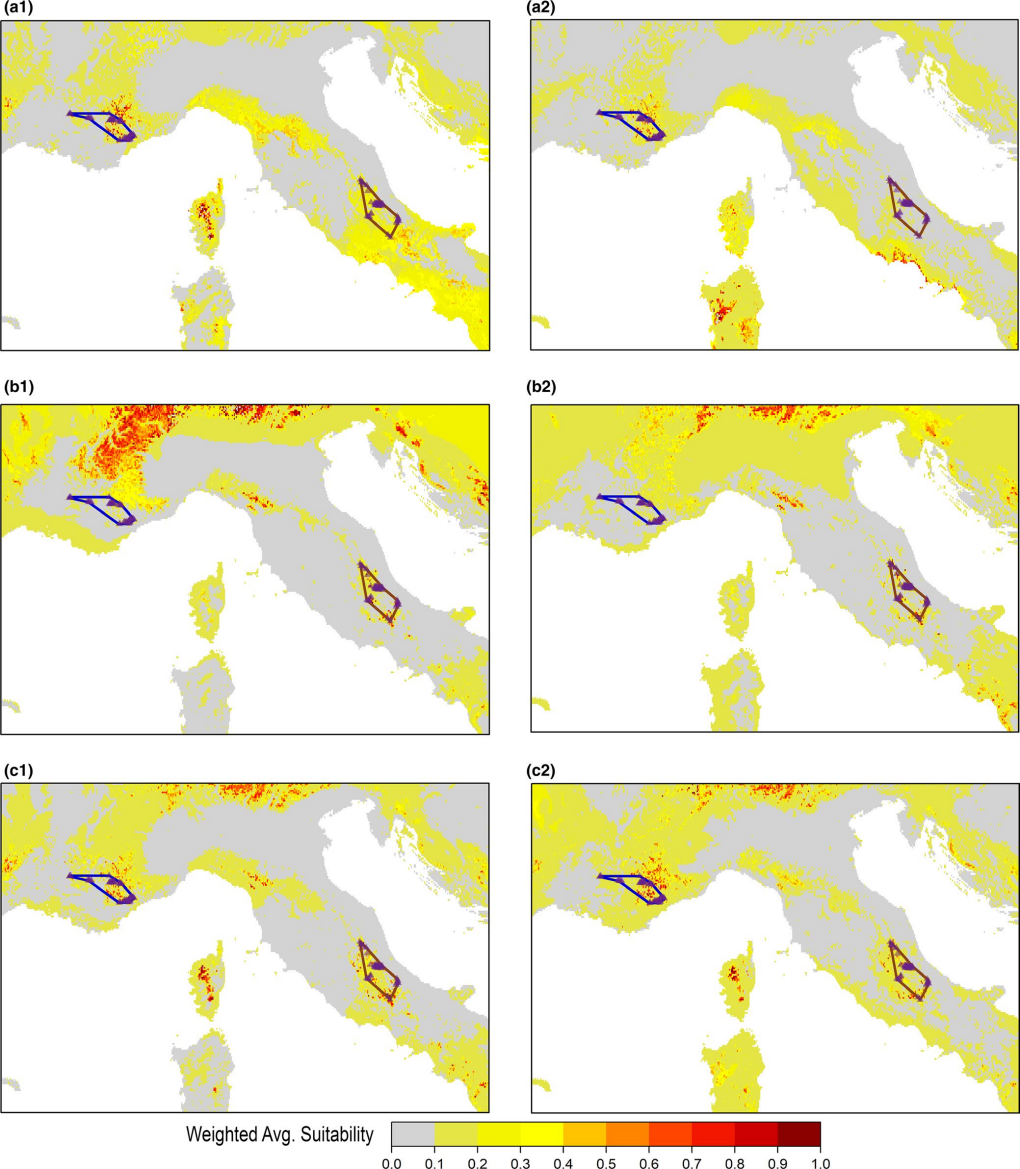
Ensemble projection of weighted average suitability derived from the HSMs fitted upon the thinned occurrence datasets, for each combination of calibration group (“France,” “Italy,” “Joint”) * set of predictors (“Ecol,” “VIF”), and obtaining Continuous Boyce Index ≥ 0.7 when validated on spatially independent test samples drawn from the calibration region (“SpBlock CV”): (a1) “France‐Ecol,” (a2) “France‐VIF,” (b1) “Italy‐Ecol,” (b2) “Italy‐VIF,” (c1) “Joint‐Ecol,” (c2) “Joint‐VIF”

Figure 6 shows weighted average suitability (Weighted_Avg_HS) predicted across the study area within the ensemble projection based on HSMs fitted on the thinned datasets for each combination of calibration group * set of predictors (“Ecol”: maps on the left; “VIF”: maps on the right); the corresponding Weighted_Avg_HS maps from HSMs fitted on the full datasets are reported in Figure S5. No major differences in suitability patterns predicted within the occurrence range of the French and Italian populations emerged between ensemble projections from “Ecol” (Figure 6a1,b1,c1) and “VIF” (Figure 6a2,b2,c2). However, wider extrapolation of highly suitable areas (Weighted_Avg_HS = 0.6–1) outside the occurrence range of the two populations’ clusters appeared for “Ecol,” especially in the ensemble projections based on HSMs fitted on the full datasets (Figure S5). Ensemble projections from the “Joint” group were the ones that best matched the overall distribution of *V. u. ursinii*, for both the thinned and the full datasets, showing several patches of highly suitable areas within the territories currently occupied by both the Italian and the French populations (Figure 6c1,c2; Figure S5c1,c2). Differently, ensemble projections from the “Italy” and “France” groups fairly modeled the current distribution of the respective populations but predicted relatively low suitability (Weighted_Avg_HS = 0.1–0.4) within the range of the geographically disjunct cluster (Figure 6a1,a2,b1,b2; Figure S5a1,a2,b1,b2).

**FIGURE 7:**
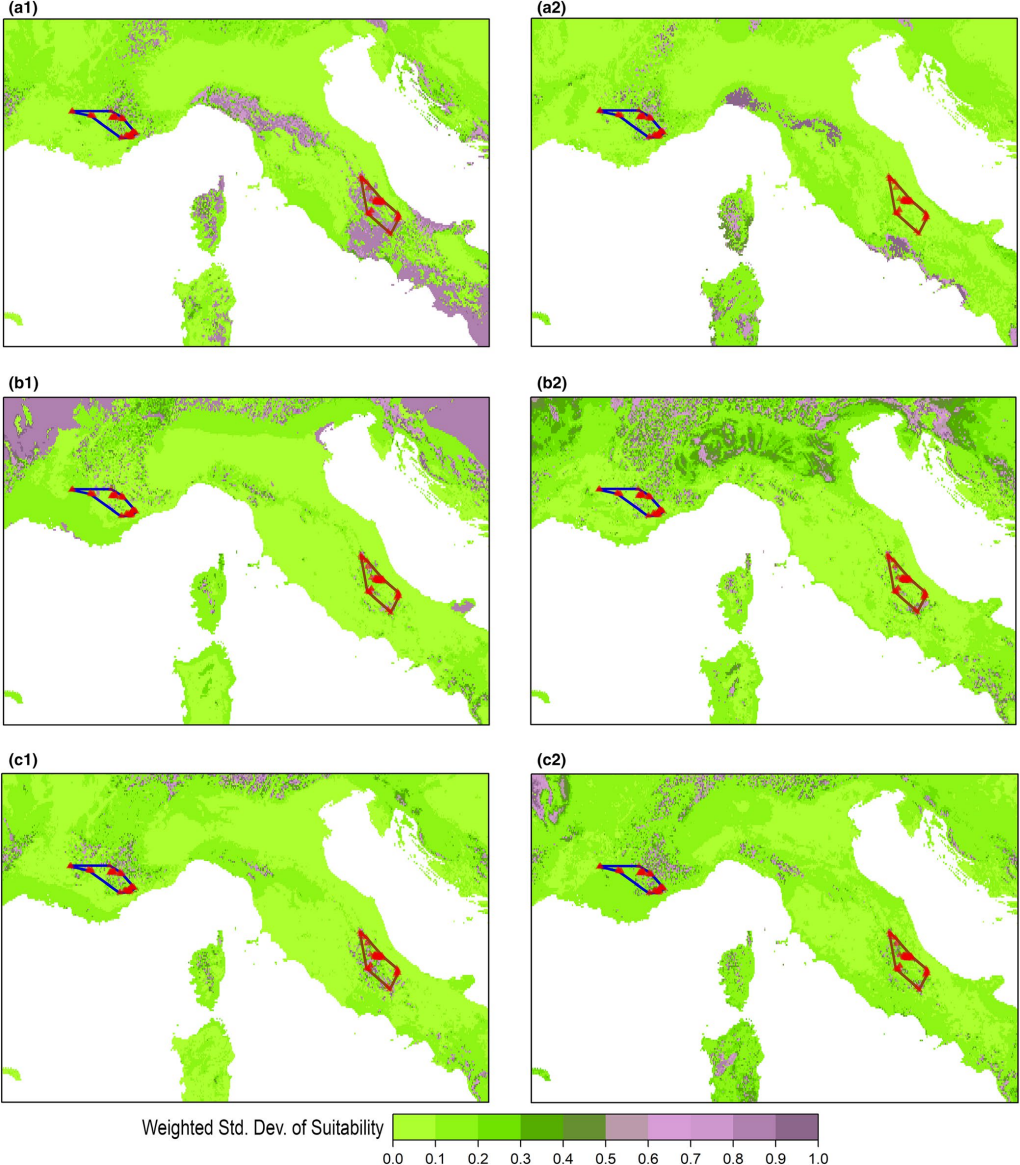
Weighted standard deviation of the suitability values predicted by the single HSMs fitted upon the thinned occurrence datasets, for each combination of calibration group (“France,” “Italy,” “Joint”) * set of predictors (“Ecol,” “VIF”), and obtaining Continuous Boyce Index ≥ 0.7 when validated on spatially independent test samples drawn from the calibration region (“SpBlock CV”): (a1) “France‐Ecol,” (a2) “France‐VIF,” (b1) “Italy‐Ecol,” (b2) “Italy‐VIF,” (c1) “Joint‐Ecol,” (c2) “Joint‐VIF”

Considering the weighted standard deviation of suitability (Weighted_StdDev_HS), ensemble projections from the “Joint” group showed lower variability than those from the “France” and “Italy” ones in suitability patterns predicted outside the range of the two populations’ clusters, particularly for the thinned datasets (Figure 7). Nonetheless, most of areas outside the French and Italian *V. u. ursinii* ranges predicted as highly suitable within ensemble projections from the “Joint” group still showed moderate‐to‐high variability among predictions of the component HSMs (i.e., Weighted_StdDev_HS > 0.5) (Figure 7c1,c2; Figure S6c1,c2). Ensemble projections from “Ecol” and “VIF” did not evidently differ in terms of Weighted_StdDev_HS, although those from HSMs fitted on the thinned datasets for the “France” and “Italy” groups showed somewhat higher variability for “Ecol” than for “VIF” outside the range of the two populations’ clusters (Figure 7a1,a2,b1,b2).

**FIGURE 8:**
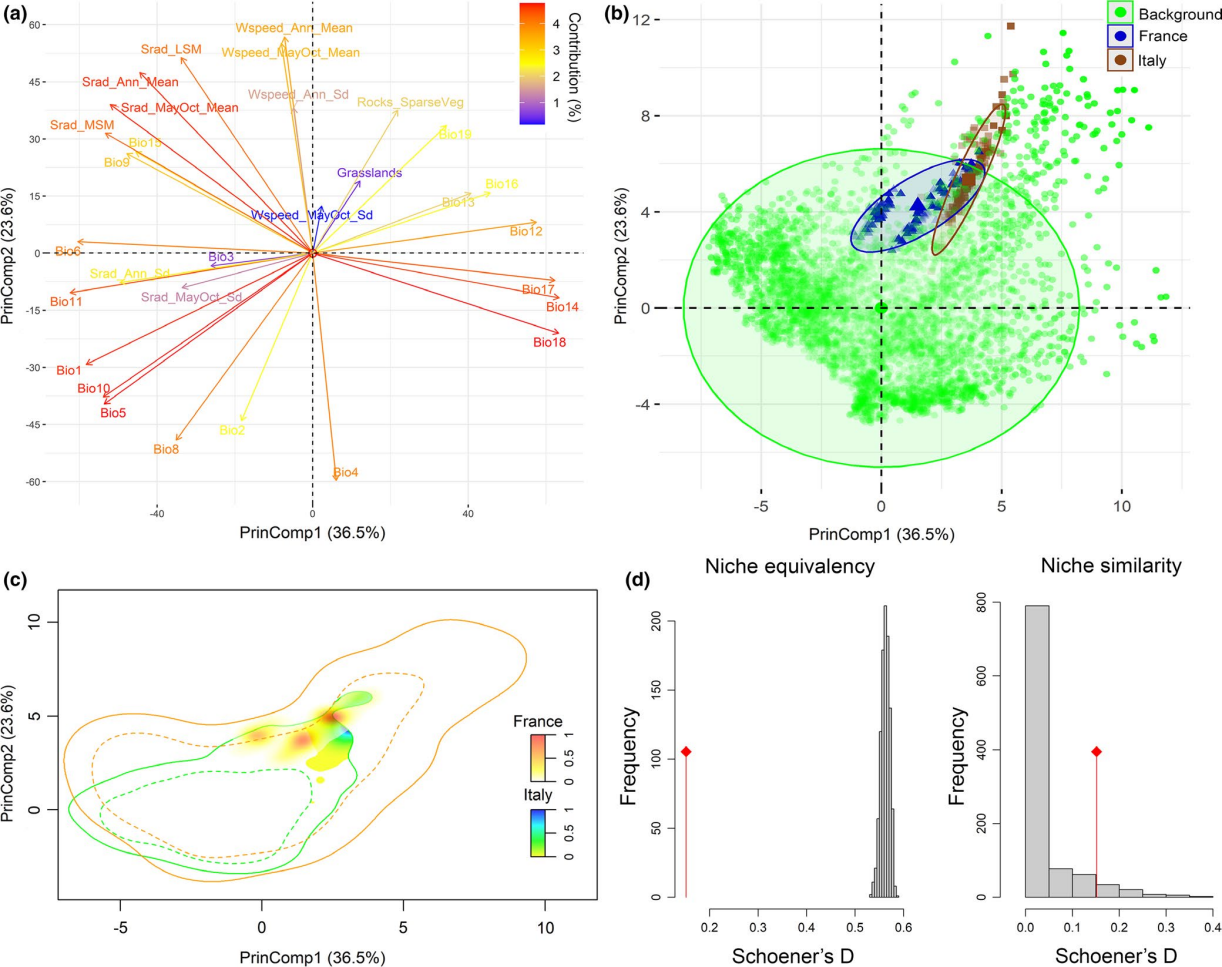
Analysis of niche overlap between the Italian and the French *Vipera ursinii ursinii* populations through the “PCA‐Env” approach. (a) contributions of the input predictors to the first two principal components resulting from the PCA; (b) position of occurrence points from the Italian (brown) and the French (blue) populations, as well as of background points used to calibrate the PCA‐Env (green), within the 2D environmental space defined by the two principal components; (c) density of occurrence of the Italian and French populations in the 2D environmental space, with solid contour lines representing the full environmental background and dashed contour lines representing 50% of the background environment; (d) simulated (histograms) and observed (red line) niche overlap *D* values compared within the niche equivalency and niche similarity (alternative hypothesis: niche divergence) tests

The PCA‐Env performed on the full set of occurrences of the Italian and French populations, considering all the 31 candidate predictors, resulted in 60.1% of the total variability explained by the first two principal components (PrinComp). Occurrences of the two populations’ clusters partially overlapped in the upper‐right quadrant of the 2D environmental space, apparently associated to the habitat‐related variables (i.e., “Grasslands” and “Rocks_SparseVeg”) and to precipitation‐related ones such as Bio13 (precipitation of the wettest month) and Bio16 (precipitation of wettest quarter) (Figure 8a,b). However, occurrences of the Italian populations were more spread along the positive half of PrinComp2, which primarily summarizes variability from variables related to wind speed patterns. The primary cores of the kernel‐smoothed densities of occurrence obtained for the French and Italian populations are noticeably close to each other (Figure 8c). Nonetheless, three separate density centers emerged for the French cluster, suggesting that the environment–occurrence relationships may vary among populations inhabiting different areas of the French range, while Italian populations primarily occupied a single restricted portion of the 2D environmental space. Moreover, environmental conditions characterizing the background area of the French populations resulted as far more diversified than those of the Italian background. Observed niche overlap between the French and the Italian populations was relatively low (*D* = 0.15). Coherently, the niche equivalency test permitted to reject the null hypothesis of their niches being equivalent (Figure 8d, *n* = 1,000, *p* = 0.001); nonetheless, the niche similarity test performed considering niche divergence as alternative hypothesis indicated that the observed overlap was not lower (*n* = 1,000, *p* = 0.93) than expected based on the background environment of the two populations’ clusters. Contrarily, niche conservatism could be accepted under a “relaxed” *α* = 0.1 according to a second niche similarity test with conservatism as alternative hypothesis (*n* = 1,000, *p* = 0.08).

The two habitat‐related variables obtained weighted average importance scores (Weighted_Avg_VarImp) higher than 5% for most of the combinations of calibration group * occurrence data size * set of predictors, but they were not comprised among the most influential predictors for any of them (Table 2). Precipitation‐related variables, particularly Bio16 and Bio15 (precipitation seasonality), were the most influential predictors for the HSMs fitted upon the French occurrences using the “Ecol” set of predictors (Table 2, Figure S7a,c). Nonetheless, inflated response curves drawn for the “France‐Ecol‐Thin” combination resulted in unclear predictor–suitability relationships (Figure S7c), probably due to the high weighted standard deviation of importance score (Weighted_StdDev_VarImp) attained by Bio16 and Bio15 (Table 2). Differently, suitability predicted from the HSMs fitted for the “France” group through the “VIF” set of predictors was positively related to annual solar radiation (Srad_Ann_Mean) and Bio13, while negatively related to variability in wind speed during *V. u. ursinii* activity period (Wspeed_MayOct_Sd) (Table 2, Figure S7b,d). Considering the HSMs fitted for the “Italy” group using the “Ecol” set, variability in solar radiation during the May–October period (Srad_MayOct_Sd), precipitation of the warmest quarter (Bio18) and mean temperature of warmest quarter (Bio10) attained the highest Weighted_Avg_VarImp, with different relative rankings depending on the considered occurrence data size (Table 2). Inflated curves drawn for these variables considering HSMs fitted upon the full datasets suggested negative association with Srad_MayOct_Sd and Bio10 (Figure S8a), while the corresponding curves from the HSMs fitted upon the thinned datasets could barely retrieve a relationship between these variables and suitability (Figure S8c). The HSMs fitted upon the Italy group using the “VIF” set isolated, for both the “Thin” and the “Full” datasets, mean temperature of the driest quarter (Bio9), annual variability of solar radiation (Srad_Ann_Sd), and Wspeed_MayOct_Sd as the most influential variables, predicting negative association between suitability and Srad_Ann_Sd, positive association with Wspeed_MayOct_Sd and unclear patterns for Bio9 (Table 2, Figure S8b,d). Finally, considering the HSMs fitted for the “Joint” group upon both the “Full” and the “Thin” datasets, Bio10 resulted as the most influential variable within the “Ecol” set, with an optimum range for suitability between 10°C and 15°C (Table 2, Figure S9a,c), while Bio9 attained the highest Weighted_Avg_VarImp within the “VIF” set, showing decreasing suitability at increasing temperatures (Table 2, Figure S9b,d).

**TABLE 2:** Weighted average percent importance score (Avg.), its standard deviation (*SD*), and the resulting coefficient of variation (Coeff. Var.) of the variables attaining Avg. ≥ 5%, for each combination of calibration group (“France,” “Italy,” “Joint”) * occurrence data size (“Full,” “Thin”) * set of predictors (“Ecol,” “VIF”)

Ecol				VIF			
Variable	Avg. (%)	*SD* (%)	Coeff. Var.	Variable	Avg. (%)	*SD* (%)	Coeff. Var.
France‐Full							
**Bio16**	**64.3**	**11**	**0.2**	**Wspeed_MayOct_Sd**	**25.2**	**6.3**	**0.2**
Wspeed_MayOct_Sd	16	8.7	0.5	**Bio13**	**24**	**6.1**	**0.3**
**Bio15**	**6.2**	**2.6**	**0.4**	**Srad_Ann_Mean**	**15.6**	**3.6**	**0.2**
**Srad_MayOct_Sd**	**6**	**1.8**	**0.3**	Bio4	9.6	4.9	0.5
/				Srad_Ann_Sd	8.1	2.5	0.3
				Bio15	7.3	2	0.3
France‐Thin							
**Bio16**	**27.8**	**24.4**	**0.9**	**Srad_Ann_Mean**	**29.7**	**9.3**	**0.3**
**Bio15**	**18.9**	**12.6**	**0.7**	Wspeed_MayOct_Sd	23.7	20	0.8
**Bio10**	**13.2**	**14.2**	**1.1**	**Bio13**	**13.2**	**5.6**	**0.4**
Bio9	10.4	23.4	2.2	**Bio15**	**11.4**	**6.3**	**0.6**
Grasslands	5.4	6.2	1.1	Rocks_SparseVeg	6.5	6.8	1
/				Bio9	5.7	11.2	2
Italy‐Full							
**Srad_MayOct_Sd**	**34**	**24.1**	**0.7**	**Bio9**	**25.4**	**23.2**	**0.9**
**Bio18**	**20.9**	**25.7**	**1.2**	**Wspeed_MayOct_Sd**	**24.6**	**27.5**	**1.1**
**Bio10**	**15.2**	**20.4**	**1.3**	**Srad_Ann_Sd**	**24.4**	**16.9**	**0.7**
Bio9	8.8	11.1	1.3	Grasslands	7	9.2	1.3
Wspeed_MayOct_Sd	7.4	18.5	2.5	Bio15	5.9	13.6	2.3
/				Srad_Ann_Mean	5.6	8.3	1.5
Italy‐Thin							
**Bio10**	**30.3**	**29.1**	**1**	**Wspeed_MayOct_Sd**	**25.8**	**23.5**	**0.9**
Bio9	15.8	23.4	1.5	**Bio9**	**23.3**	**24.3**	**1**
**Srad_MayOct_Sd**	**10.9**	**13.4**	**1.2**	**Srad_Ann_Sd**	**21.2**	**26**	**1.2**
**Bio18**	**10**	**10**	**1**	Grasslands	10.1	12.8	1.3
Wspeed_MayOct_Sd	8.4	18.1	2.2	Srad_Ann_Mean	6.8	21.1	3.1
Grasslands	6.5	7.8	1.2	Rocks_SparseVeg	5.1	8.8	1.7
Bio2	5.1	7.5	1.5	/			
Joint‐Full							
**Bio10**	**35.1**	**26.1**	**0.7**	**Bio9**	**22.4**	**11**	**0.5**
Bio16	18.1	17.7	1	**Wspeed_MayOct_Sd**	**18.9**	**13.7**	**0.7**
**Wspeed_MayOct_Mean**	**10.7**	**4.8**	**0.4**	Srad_Ann_Mean	15.6	12.9	0.8
Grasslands	7.1	8.4	1.2	**Bio15**	**15.3**	**7.2**	**0.5**
**Wspeed_Ann_Sd**	**6.8**	**3.9**	**0.6**	Grasslands	8.7	11.3	1.3
Bio15	6.5	7.8	1.2	Bio13	6.9	11.7	1.7
Joint‐Thin							
**Bio10**	**31.3**	**20.2**	**0.6**	**Bio9**	**25.5**	**13.3**	**0.5**
Bio18	9.4	17.2	1.8	**Bio15**	**16**	**18.3**	**1.1**
**Bio9**	**8.8**	**6.2**	**0.7**	Wspeed_MayOct_Sd	12.4	14.7	1.2
**Srad_MayOct_Sd**	**8.2**	**6.7**	**0.8**	**Srad_Ann_Mean**	**10**	**8**	**0.8**
Grasslands	7.6	8.3	1.1	Grasslands	7.5	8.2	1.1
Wspeed_MayOct_Mean	6.7	5.6	0.8	Rocks_SparseVeg	7.3	6	0.8
Srad_MayOct_Mean	5.3	5.3	1	Bio4	6.9	7	1
Bio15	5	4.2	0.8	Bio13	5.9	4.9	0.8
/				Bio3	5.2	5	1

Note

Weights applied to the importance scores assigned to the variables within each HSM correspond to the Continuous Boyce Index value the model attained when validated on spatially independent test samples drawn from the calibration region (“SpBlock CV”). Variables in bold show the three lowest coefficients of variation of importance score among the ones attaining the five highest Avg. values.

## 4 DISCUSSION

Transferability of HSMs represents a compelling challenge when predictions to new spatiotemporal scenarios are required (Franklin, 2013; Yates et al., 2018). Our modeling framework permitted to investigate the potential influence of several factors upon HSMs transferability within and between the French and Italian disjunct populations’ clusters of *V. u. ursinii*.

*Vipera u. ursinii* is a mountain‐adapted subspecies of meadow viper showing a peculiar phenology: Adults interrupt hibernation in late April for the breeding season and juveniles emerge later in early summer for feeding; both adults and juveniles usually return to wintering shelters by the end of September in the subspecies’ Italian range (Luiselli, 2004), while the activity period may extend until October–November in the French range (Lisse et al., 2012). The known association of *V. u. ursinii* with typical mountainous habitats such as grasslands and rocky areas (Luiselli, 2004; Lyet et al., 2013) was retrieved for both the French and the Italian populations in the respective niche occupancy modeled through the PCA‐env approach (Figure 8a,b). On the other hand, percent cover of grasslands or rocky and sparsely vegetated areas were not selected as critical predictors for the environment–occurrence relationships estimated by the HSMs. Contrarily, different sets of climate‐related predictors attained the highest weighted importance scores depending on the considered combination of calibration group * occurrence data size * set of predictors (Table 2). The HSMs fitted upon occurrences from the whole *V. u. ursinii* known distribution (i.e., the “Joint” group) mainly retrieved temperature of the driest (Bio9) and warmest (Bio10) quarter as driving predictors; differently, the models separately fitted for the two populations’ clusters returned different mixes of climate‐related predictors influencing suitability. These results agree with previous evidences of temperature trends shaping the environmental niche of herptiles across wide geographical extents (Araújo et al., 2006; Guisan & Hofer, 2003), while precipitation, solar radiation, and wind speed emerge as important predictors when modeling herptiles’ suitability at more local scale due to their influence on thermoregulation and other critical factors such as prey availability (Mizsei et al., 2016; Ortega et al., 2017). Moreover, the fact that the obtained HSMs primarily correlated suitability for *V. u. ursinii* to climatic patterns rather than to the habitat‐related variables echoes previous findings suggesting that, once the important climatic drivers are included, HSMs predictions at the meso‐ to macroscale may not benefit from the addition of predictors representing land cover or vegetation types (Bucklin et al., 2015; Thuiller et al., 2004). Nonetheless, the upscaling of information about the percent cover of grasslands or rocky and sparsely vegetated areas from the original 100 m resolution to that of the climate‐related predictors may have hidden part of the fine‐scale association between *V. u. ursinii* and such habitat types found in previous modeling studies conducted upon the French populations (Lyet et al., 2013). The most proximal drivers of *V. u. ursinii* presence and activity patterns could be better understood gaining additional information from mechanistic models relying on experimental studies targeting its ecophysiology, but this is beyond the scope of the present work.

The realized niche of the Italian *V. u. ursinii* populations emerged as narrower than that of the French ones, which also showed background environmental conditions extending far beyond those characterizing the Italian range (Figure 8c). The wider niche occupancy and background of the French populations may reflect the fact that areas of southeastern France hosting *V. u. ursinii* are located at the boundaries between the Alpine and the Mediterranean biogeographic regions: the corresponding “hybrid” climate may in turn determine more diversified environmental conditions. Despite the low observed niche overlap between the two populations’ clusters, the performed similarity test did not corroborate niche divergence while niche conservatism emerged as more probable. This may reflect the fact that, once *V. u. ursinii* distribution contracted after the last glacial maximum due to the disappearance of lower mountainous grasslands following the upshift of tree line (Ferchaud et al., 2012), the remnant Italian and French populations, given the low dispersal capability of this meadow viper (Lisse et al., 2012), were confined to areas hosting different environmental conditions but comprised within the species’ fundamental niche. Similar patterns of current allopatric distributions, shaped by paleoclimatic events, without significant niche divergence among geographically disjunct lineages despite differences in the realized environments characterizing their respective occurrence areas were recently found for *Vipera aspis* and *Vipera latastei* (Martínez‐Freiría et al., 2020).

The two approaches to predictors filtering we tested did not lead to significant differences in terms of internal or external transferability. Nonetheless, the inflated response curves drawn for the predictors attaining high weighted importance scores indicated somewhat discrepant environment–occurrence relationships between the HSMs fitted through the “Ecol” set of predictors and those fitted through the “VIF” one (Figures S7–S9). Moreover, ensemble projections derived from the two sets of predictors contrasted in suitability patterns predicted outside the current range of the French and Italian populations (Figures 6, 7; Figures S5, S6). This confirms that HSMs fitted upon the same data using different predictors may produce discrepant spatial predictions while maintaining similar predictive capabilities (Bucklin et al., 2015).

Recent studies targeting the relative ranking of several algorithms in terms of transferability showed good performances for both GAM and GBM (Duque‐Lazo et al., 2016; Heikkinen et al., 2012; Qiao et al., 2019). The higher AUC attained by GBM within our modeling framework may be due to its capacity to reach higher specificity than GAM in both interpolation and extrapolation tasks (Heikkinen et al., 2012; Qiao et al., 2019), tough specificity could not be properly estimated here as we used pseudoabsences instead of “true” absences. Moreover, GBM is claimed to provide a good trade‐off between complexity of the estimated environment–occurrence relationships and avoidance of overfitting (Elith & Graham, 2009; Heikkinen et al., 2012); this could explain the fact that GBM obtained higher Continuous Boyce Index and was less sensitive to multivariate novelty than GAM when HSMs were validated on spatially independent test samples from the calibration region (“SpBlock CV”). Differently, GAM outperformed GBM when HSMs were transferred on the geographically disjunct populations’ cluster (“External validation”), echoing results from Duque‐Lazo et al. (2016). All in all, it should be noted that discrepant rankings of the single algorithms in transferability tasks among different studies may also derive from the way algorithms were parameterized and the resulting HSMs evaluated (e.g., spatial blocking cross‐validation versus repeated split‐sample), as highlighted in Yates et al. (2018).

Spatial thinning of occurrence data ameliorated internal transferability for all the three calibration groups according to Continuous Boyce Index scores in “SpBlock CV”, corroborating the usefulness of rarefying geographically close occurrences when sampling biases in data collection could not be a priori excluded (Boria et al., 2014). The fact that the “France” group was the one showing the worst performance in internal transferability and at the same time the one benefitting the most from spatial thinning is worth of attention. The “France” group underwent the highest proportional reduction in the number of occurrences from the “Full” dataset to the “Thin” one; contextually, environmental heterogeneity within *V. u. ursinii* French range was clearly more pronounced than within the Italian one (Figure 8c): Reducing the influence of possible sampling biases as well as the novel combinations of environmental conditions in test sites, spatial thinning eased interpolation for the HSMs fitted on the French occurrences. This “environmental noise” emerging from the French datasets probably also made HSMs from the “Joint” group performing worse than those from the “Italy” one in internal transferability, as it increased the difficulty for the HSMs to project the environment–occurrence relationships fitted on the training fold to the environmentally heterogeneous test fold derived from checkerboard blocking (Roberts et al., 2017).

On the other hand, spatial thinning did not provide beneficial effects for external transferability, and the HSMs separately fitted upon the French and the Italian occurrences showed consistently lower predictive performance in “External validation” than in “SpBlock CV”. This echoes results from previous studies about HSMs’ internal versus external transferability (Barbosa et al., 2009; Duque‐Lazo et al., 2016; Randin et al., 2006) and suggests that transferability across wide geographical extents is more related to the environmental novelty of test data than to the number and spatial arrangement of calibration data. Indeed, validation on test localities far from the calibration area increases the probability of HSMs being projected on conditions significantly different from those upon which they fitted the environment–occurrence relationships, forcing them to extrapolate (Qiao et al., 2019). Our finding that the degree of multivariate extrapolation, which was expectedly higher in “External validation” than in “SpBlock CV,” was significantly related to the decrease in predictive performance considering both AUC and Continuous Boyce Index further strengthens this hypothesis.

Weighted ensemble projections derived from the HSMs fitted on the “Joint” group, considering both the “Full” and “Thin” Pres‐PseudoAbs datasets, correctly predicted as suitable the patches currently occupied by *V. u. ursinii* within both its French and Italian range. Contrarily, ensemble projections from HSMs fitted on partial Pres‐PseudoAbs datasets did not properly predict suitable areas within the geographically disjunct range not considered for model calibration. Moreover, ensemble projections from the Joint datasets showed lower variability among the component HSMs than ensemble projections separately obtained for the two populations’ clusters, especially outside the occurrence areas of these latter. These trends confirm that spatial predictions of suitability benefit from calibration data covering as much as possible the range of environmental conditions the target entity experiences (Qiao et al., 2019), particularly when intraspecific niche differences at the local scale are presumed to exist (Barbosa et al., 2009; Carretero & Sillero, 2016).

In conclusion, we could summarize our findings about spatial transferability of HSMs at intraspecific level as follows: (a) In case the aim is to get predictions within the surroundings of a populations’ cluster, for instance to individuate promising sites looking for unknown populations, spatial thinning of occurrence data is recommended, particularly when noticeable environmental heterogeneity characterizes the calibration area; (b) behavior of different algorithms in interpolation and extrapolation tasks should be taken into account when data from extensively surveyed populations are used to estimate the environment–occurrence relationships and then investigate the distribution of suitable sites for distant, poorly known populations; (c) the environment–occurrence relationships estimated by the HSMs, and the resulting spatial predictions of suitability, should be critically examined also in light of the selected set of predictors; (d) the risk of projecting HSMs on new regions should be a priori evaluated based on the degree of multivariate environmental novelty of projection areas, which may be considerable when geographically segregated intraspecific lineages occur in areas hosting different environmental conditions and HSMs are calibrated upon data covering a subset of the species’ realized niche. Future studies investigating the effect of finer‐scale environmental drivers and biotic correlates of populations’ persistence (Ficetola et al., 2018; Peñalver‐Alcázar et al., 2016) on niche dynamics of geographically disjunct populations would surely help in further clarifying these and additional determinants of spatial transferability.

## CONFLICT OF INTEREST

The authors declare no conflict of interest.

## AUTHOR CONTRIBUTIONS

**Francesco Cerasoli:** Conceptualization (lead); data curation (lead); formal analysis (lead); investigation (equal); methodology (lead); writing – original draft (equal); writing – review and editing (equal). **Aurélien Besnard:** Conceptualization (lead); data curation (lead); formal analysis (supporting); investigation (equal); methodology (supporting); writing – original draft (equal); writing – review and editing (equal). **Marc‐Antoine Marchand:** Conceptualization (lead); data curation (lead); formal analysis (supporting); investigation (equal); methodology (supporting); writing – original draft (equal); writing – review and editing (equal). **Paola D'Alessandro:** Conceptualization (supporting); data curation (supporting); formal analysis (supporting); investigation (equal); methodology (supporting); writing – original draft (equal); writing – review and editing (equal). **Mattia Iannella:** Conceptualization (supporting); data curation (lead); formal analysis (supporting); investigation (equal); methodology (supporting); writing – original draft (equal); writing – writing – review and editing (equal). **Maurizio Biondi:** Conceptualization (lead); data curation (supporting); formal analysis (supporting); investigation (equal); methodology (supporting); writing – original draft (equal); writing – review and editing (equal).

## Supporting information

Supplementary Material

Supplementary Material

## Data Availability

Since *Vipera ursinii* represents a highly threatened species, classified as “Vulnerable” by the IUCN, we prefer not to share exact coordinates of occurrence localities upon publicly accessible repositories (Lunghi et al., 2019). However, exact coordinates of Italian and French *V. ursinii ursinii* occurrences gathered from Console et al. (2020), as well as the R code written to conduct all the analyses, are freely available from authors upon motivated request. The French occurrence data obtained from SILENE cannot be stored on public repositories as well, because the agreement signed with SILENE does not allow the authors to share the data, but they are freely available at the highest resolution upon request (and freely downloadable at 5 km^2^ resolution) at www.silene.eu.
