# Supplementary material for: Determinants of habitat suitability models transferability across geographically disjunct populations: Insights from Vipera ursinii urs inii
Source: Ecol Evol. 2021 Mar 17;11(9):3991–4011. doi: 10.1002/ece3.7294 (PMC8093743; doi:10.1002/ece3.7294)

**Appendix**

**
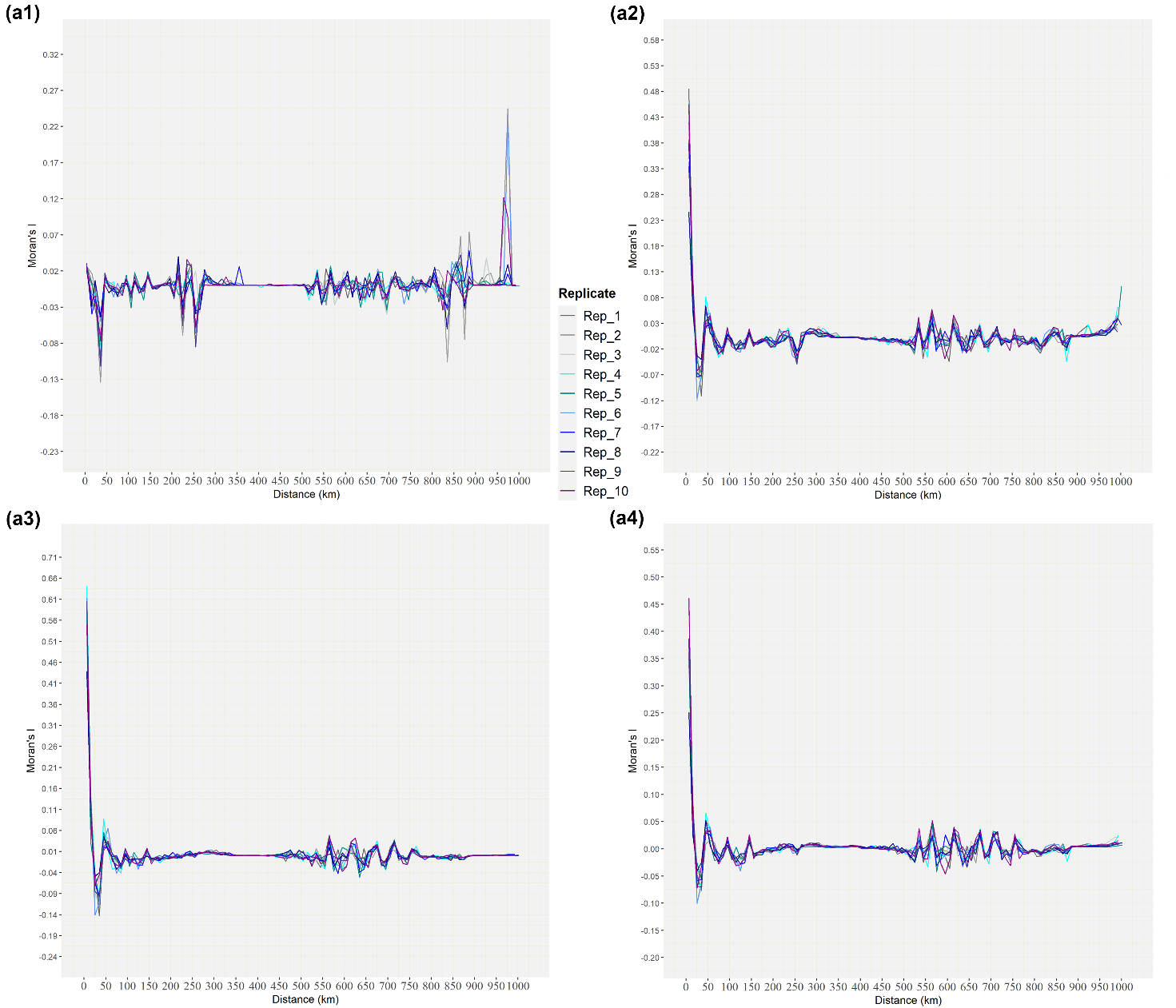
Figure S1.** Correlograms showing Moran’s Index (I), computed on residuals from the predictions of the initial HSMs on the training data, at increasing inter-point distances (pace = 10 km). (a1) GAM models fitted on the ‘Joint-Thin’ combination using the ‘Ecol’ set of predictors; (a2) GBM models fitted on the ‘Joint-Thin’ combination using the ‘Ecol’ set of predictors; (a3) GAM models fitted on the ‘Joint-Thin’ combination using the ‘VIF’ set of predictors; (a4) GBM models fitted on the ‘Joint-Thin’ combination using the ‘VIF’ set of predictors;

**Figure S1. (continues from previous page)** (b1) GAM models fitted on the ‘Joint-Full’ combination using the ‘Ecol’ set of predictors; (b2) GBM models fitted on the ‘Joint- Full’ combination using the ‘Ecol’ set of predictors; (b3) GAM models fitted on the ‘Joint- Full’ combination using the ‘VIF’ set of predictors; (b4) GBM models fitted on the ‘Joint- Full’ combination using the ‘VIF’ set of predictors**.**

**
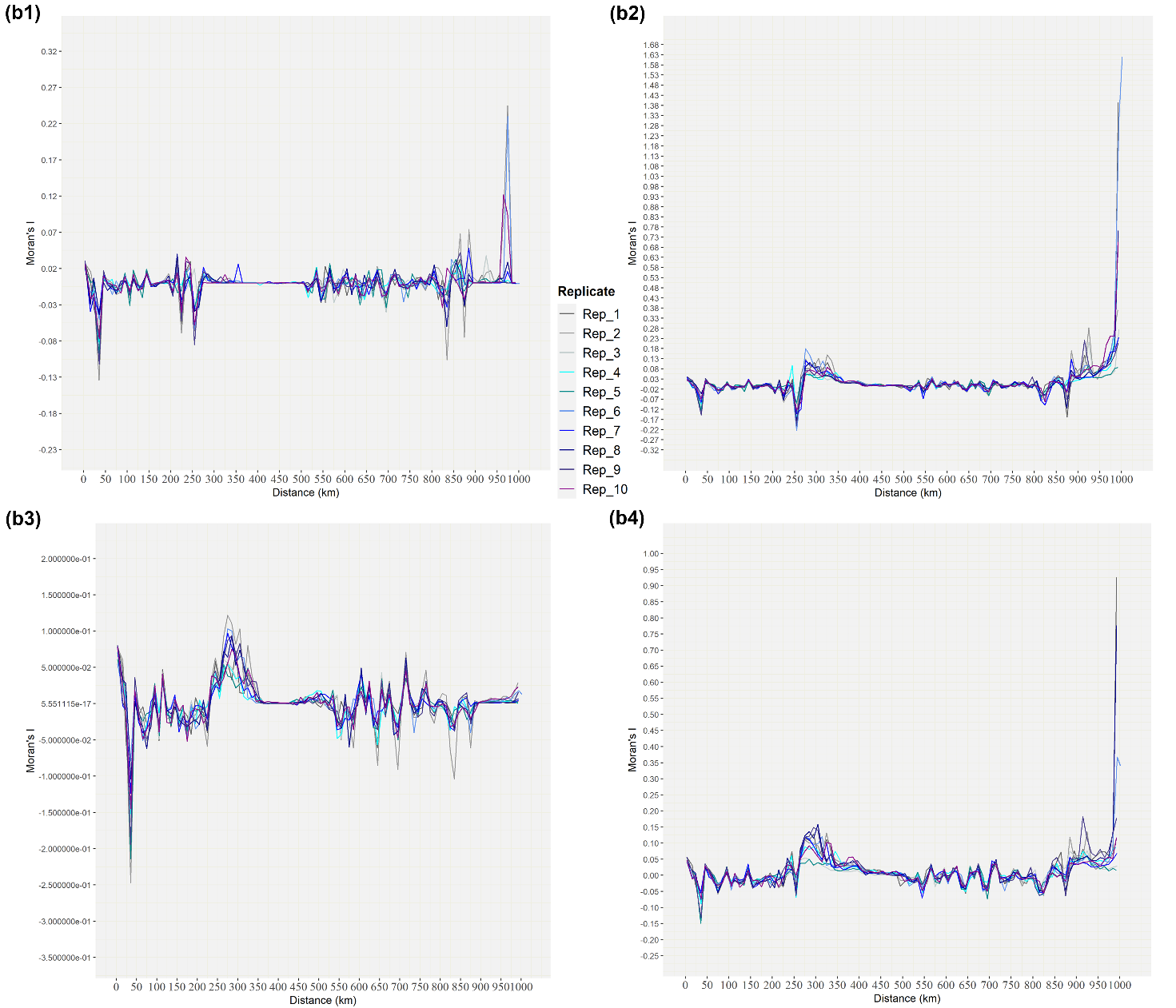
**

**
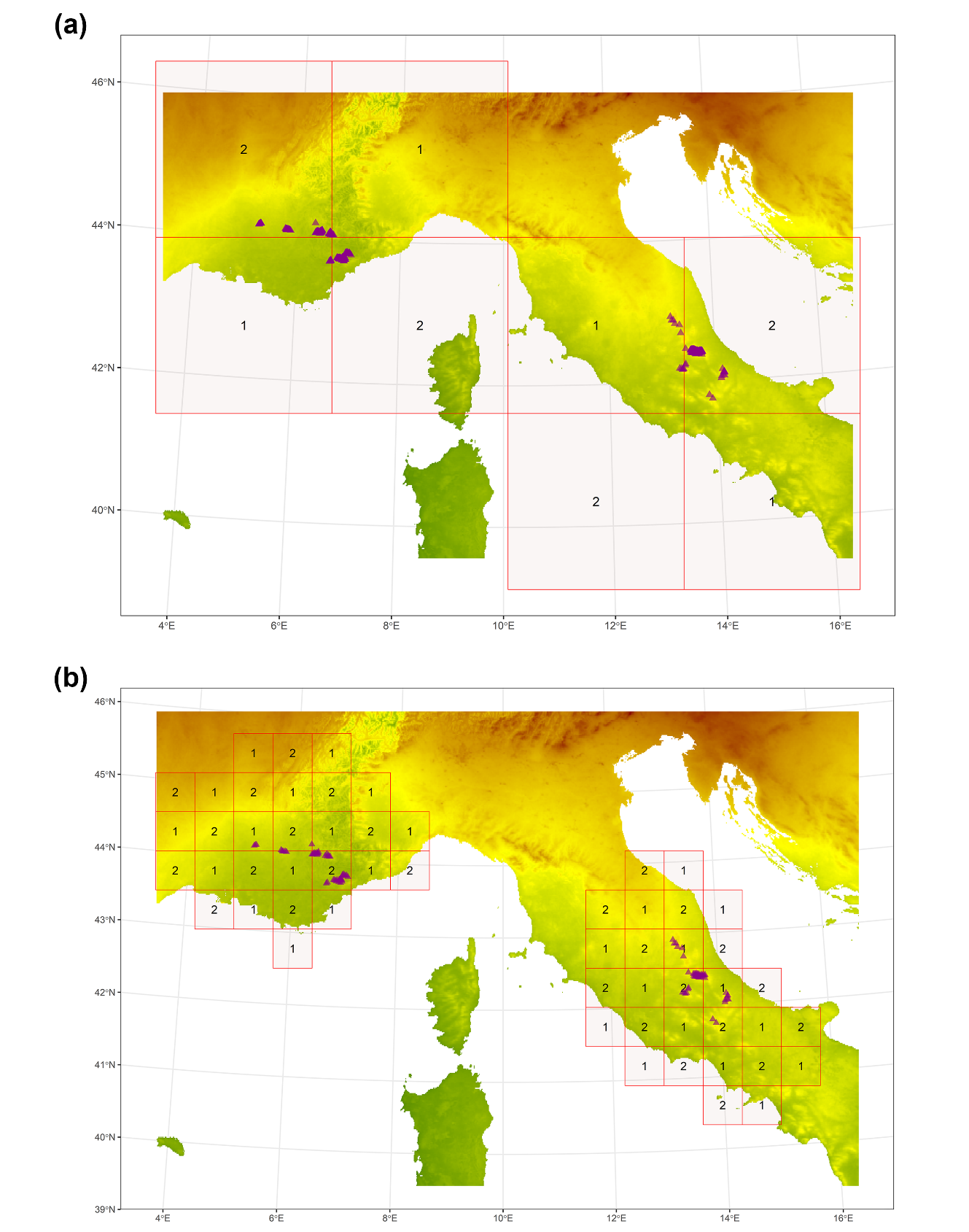
Figure S2.** Example maps showing the distribution of the (a) ‘Full’ and (b) ‘Thin’ *V. u. ursinii* occurrences (purple triangles) within the checkerboard spatial blocks arranged for the ‘Joint’ calibration group, based on patterns of spatial autocorrelation emerging from correlograms on HSMs residuals. Block size was 275 km for the ‘Joint-Full’ combination and 60 km for the ‘Joint-Thin’ one. Numbers within the blocks correspond to the blocks-to-fold checkerboard assignment

**Table S1.** Summary statistics of the linear models relating, for each combination of calibration group (‘France’, ‘Italy’, ‘Joint’) * occurrence data size (‘Full’, ‘Thin’), AUC scores attained by the HSMs in ‘SpBlock CV’ to the algorithm (GAM, GBM) and set of predictors (‘Ecol’, ‘VIF’) used to fit the models. Rows in bold correspond to significant effect (at α = 0.05) of a factor or of interactions.

| *AUC ~ Algorithm * Set of predictors* | | | | |
| --- | --- | --- | --- | --- |
| **France - Full** | | | | |
|  | estimate | std. err. | *t* | *p* |
| **Intercept (GAM _Ecol)** | **0,830** | **0,029** | **28,991** | **< 0,001** |
| **GBM** | **0,109** | **0,040** | **2,682** | **0,0090** |
| VIF | -0,048 | 0,040 | -1,196 | 0,2355 |
| GBM*VIF | 0,057 | 0,057 | 1,003 | 0,3192 |
| **France - Thin** | | | | |
|  | estimate | std. err. | *t* | *p* |
| **Intercept (GAM_Ecol)** | **0,792** | **0,032** | **24,525** | **< 0,001** |
| **GBM** | **0,092** | **0,046** | **2,013** | **0,0477** |
| VIF | -0,014 | 0,046 | -0,300 | 0,7649 |
| GBM*VIF | 0,011 | 0,065 | 0,170 | 0,8652 |
| **Italy - Full** | | | | |
|  | estimate | std. err. | *t* | *p* |
| **Intercept (GAM_Ecol)** | **0,948** | **0,009** | **104,290** | **< 0,001** |
| **GBM** | **0,047** | **0,013** | **3,655** | **< 0,001** |
| VIF | 0,021 | 0,013 | 1,629 | 0,1074 |
| GBM*VIF | -0,024 | 0,018 | -1,298 | 0,1983 |
| **Italy- Thin** | | | | |
|  | estimate | std. err. | *t* | *p* |
| **Intercept (GAM_Ecol)** | **0,959** | **0,006** | **157,564** | **< 0,001** |
| **GBM** | **0,026** | **0,009** | **3,043** | **0,0032** |
| VIF | 0,004 | 0,009 | 0,499 | 0,6190 |
| GBM*VIF | -0,004 | 0,012 | -0,349 | 0,7281 |
| **Joint - Full** | | | | |
|  | estimate | std. err. | *t* | *p* |
| **Intercept (GAM_Ecol)** | **0,957** | **0,007** | **131,661** | **< 0,001** |
| GBM | 0,013 | 0,010 | 1,289 | 0,2012 |
| **VIF** | **-0,020** | **0,010** | **-1,956** | **0,0541** |
| GBM*VIF | 0,026 | 0,015 | 1,765 | 0,0816 |
| **Joint - Thin** | | | | |
|  | estimate | std. err. | *t* | *p* |
| **Intercept (GAM_Ecol)** | **0,930** | **0,005** | **189,041** | **< 0,001** |
| **GBM** | **0,024** | **0,007** | **3,435** | **0,0010** |
| VIF | -0,006 | 0,007 | -0,884 | 0,3795 |
| **GBM*VIF** | **-0,020** | **0,010** | **-2,043** | **0,0445** |

**Table S2.** Summary statistics of the linear models relating Continuous Boyce Index (*B*) scores attained by the HSMs in ‘SpBlock CV’ to calibration group (‘France’, ‘Italy’, ‘Joint’), occurrence data size (‘Full’, ‘Thin’), set of predictors (‘Ecol’, ‘VIF’) and algorithm (GAM, GBM) used to fit the models. Rows in bold correspond to significant effect (at α = 0.05) of a factor or of interactions.

| *B ~ Calibration group + Occurrence data size + Set of predictors + Algorithm* | | | | |
| --- | --- | --- | --- | --- |
|  | estimate | std. err. | *t* | *p* |
| **Intercept (France_Full_Ecol_GAM)** | **0,189** | **0,037** | **5,167** | **< 0,001** |
| **Italy** | **0,248** | **0,036** | **6,902** | **< 0,001** |
| **Joint** | **0,157** | **0,036** | **4,386** | **< 0,001** |
| **Thin** | **0,165** | **0,029** | **5,672** | **< 0,001** |
| VIF | 0,021 | 0,029 | 0,735 | 0,4625 |
| **GBM** | **0,216** | **0,029** | **7,420** | **< 0,001** |
| *B ~ Calibration group * Occurrence data size + Algorithm* | | | | |
|  | estimate | std. err. | *t* | *p* |
| **Intercept (France_Full_GAM)** | **0,119** | **0,038** | **3,123** | **0,0019** |
| **Italy** | **0,399** | **0,050** | **8,037** | **< 0,001** |
| **Joint** | **0,257** | **0,049** | **5,241** | **< 0,001** |
| **Thin** | **0,340** | **0,051** | **6,724** | **< 0,001** |
| **GBM** | **0,212** | **0,029** | **7,428** | **< 0,001** |
| **Italy*Thin** | **-0,306** | **0,071** | **-4,333** | **< 0,001** |
| **Joint*Thin** | **-0,209** | **0,070** | **-2,977** | **0,0031** |

**Table S3.** Summary statistics of the linear models relating, for each combination of calibration group (‘France’, ‘Italy’) * occurrence data size (‘Full’, ‘Thin’), AUC scores attained by the HSMs in ‘External validation’ to the algorithm (GAM, GBM) and set of predictors (‘Ecol’, ‘VIF’) used to fit the models. Rows in bold correspond to significant effect (at α = 0.05) of a factor or of interactions.

| *AUC ~ Algorithm * Set of predictors* | | | | |
| --- | --- | --- | --- | --- |
| **France - Full** | | | | |
|  | estimate | std. err. | *t* | *p* |
| **Intercept (GAM_Ecol)** | **0,685** | **0,025** | **27,816** | **< 0,001** |
| **GBM** | **0,070** | **0,035** | **2,022** | **0,0467** |
| **VIF** | **-0,078** | **0,035** | **-2,233** | **0,0285** |
| GBM*VIF | -0,017 | 0,049 | -0,349 | 0,7279 |
| **France - Thin** | | | | |
|  | estimate | std. err. | *t* | *p* |
| **Intercept (GAM_Ecol)** | **0,606** | **0,023** | **25,821** | **< 0,001** |
| **GBM** | **0,104** | **0,033** | **3,125** | **0,0025** |
| VIF | -0,004 | 0,033 | -0,127 | 0,8997 |
| GBM*VIF | -0,061 | 0,047 | -1,291 | 0,2007 |
| **Italy - Full** | | | | |
|  | estimate | std. err. | *t* | *p* |
| **Intercept (GAM_Ecol)** | **0,613** | **0,020** | **30,697** | **< 0,001** |
| **GBM** | **0,169** | **0,028** | **5,962** | **< 0,001** |
| **VIF** | **0,074** | **0,028** | **2,611** | **0,0109** |
| **GBM*VIF** | **-0,131** | **0,040** | **-3,266** | **0,0016** |
| **Italy- Thin** | | | | |
|  | estimate | std. err. | *t* | *p* |
| **Intercept (GAM_Ecol)** | **0,717** | **0,016** | **43,784** | **< 0,001** |
| **GBM** | **0,061** | **0,023** | **2,627** | **0,0104** |
| VIF | -0,002 | 0,023 | -0,095 | 0,9245 |
| GBM*VIF | -0,048 | 0,033 | -1,459 | 0,1487 |

**Table S4.** Summary statistics of the linear models relating Continuous Boyce Index (*B*) scores attained by the HSMs in ‘External validation’ to calibration group (‘France’, ‘Italy’), occurrence data size (‘Full’, ‘Thin’), set of predictors (‘Ecol’, ‘VIF’) and algorithm (GAM, GBM) used to fit the models. Rows in bold correspond to significant effect (at α = 0.05) of a factor or of interactions.

| *B ~ Calibration group + Occurrence data size + Set of predictors + Algorithm* | | | | |
| --- | --- | --- | --- | --- |
|  | estimate | std. err. | *t* | *p* |
| **Intercept (France_Full_Ecol_GAM)** | **-0,339** | **0,142** | **-2,384** | **0,0184** |
| **Italy** | **0,332** | **0,112** | **2,949** | **0,0037** |
| Thin | 0,082 | 0,096 | 0,857 | 0,3929 |
| **VIF** | **0,237** | **0,096** | **2,457** | **0,0151** |
| **GBM** | **-0,240** | **0,102** | **-2,346** | **0,0203** |
| *B ~ Calibration group + Algorithm * Set of predictors* | | | | |
|  | estimate | std. err. | *t* | *p* |
| Intercept (France_GAM_Ecol) | -0,111 | 0,159 | -0,695 | 0,4880 |
| **Italy** | **0,314** | **0,112** | **2,816** | **0,0055** |
| **GBM** | **-0,469** | **0,150** | **-3,127** | **0,0021** |
| VIF | -0,037 | 0,165 | -0,224 | 0,8229 |
| **GBM*VIF** | **0,398** | **0,203** | **1,959** | **0,0519** |

**Table S5.** Summary statistics of the linear models relating AUC, for each combination of calibration group (‘France’, ‘Italy’, ‘Joint’) * occurrence data size (‘Full’, ‘Thin’), and Continuous Boyce Index (*B*) across all the other investigated factors, to the validation approach (‘SpBlock CV’, ‘External’) used to test transferability.

| *AUC ~ Validation approach* | | | | |
| --- | --- | --- | --- | --- |
| **France - Full** | | | | |
|  | estimate | std. err. | *t* | *p* |
| **Intercept (External validation)** | **0,677** | **0,015** | **45,534** | **< 0,001** |
| **SpBlock CV** | **0,197** | **0,021** | **9,367** | **< 0,001** |
| **Italy - Full** | | | | |
|  | estimate | std. err. | *t* | *p* |
| **Intercept (External validation)** | **0,702** | **0,009** | **76,592** | **< 0,001** |
| **SpBlock CV** | **0,274** | **0,013** | **21,172** | **< 0,001** |
| **France - Thin** | | | | |
|  | estimate | std. err. | *t* | *p* |
| **Intercept (External validation)** | **0,641** | **0,015** | **43,338** | **< 0,001** |
| **SpBlock CV** | **0,193** | **0,021** | **9,219** | **< 0,001** |
| **Italy - Thin** | | | | |
|  | estimate | std. err. | *t* | *p* |
| **Intercept (External validation)** | **0,734** | **0,006** | **113,607** | **< 0,001** |
| **SpBlock CV** | **0,240** | **0,009** | **26,229** | **< 0,001** |
|  | | | | |
| *B ~ Validation approach* | | | | |
|  | estimate | std. err. | *t* | *p* |
| **Intercept (External validation)** | **-0,085** | **0,038** | **-2,211** | **0,0275** |
| **SpBlock CV** | **0,608** | **0,047** | **12,855** | **< 0,001** |

**Figure S3.** Scatterplot showing variation in AUC scores, for each combination of calibration group (‘France’, ‘Italy’) * occurrence data size (‘Full’, ‘Thin’), attained by the HSMs fitted through the GAM (circles) and GBM (triangles) algorithms at increasing level of multivariate novelty in test data, within ‘SpBlock CV’ (purple) and ‘External validation’ (green).


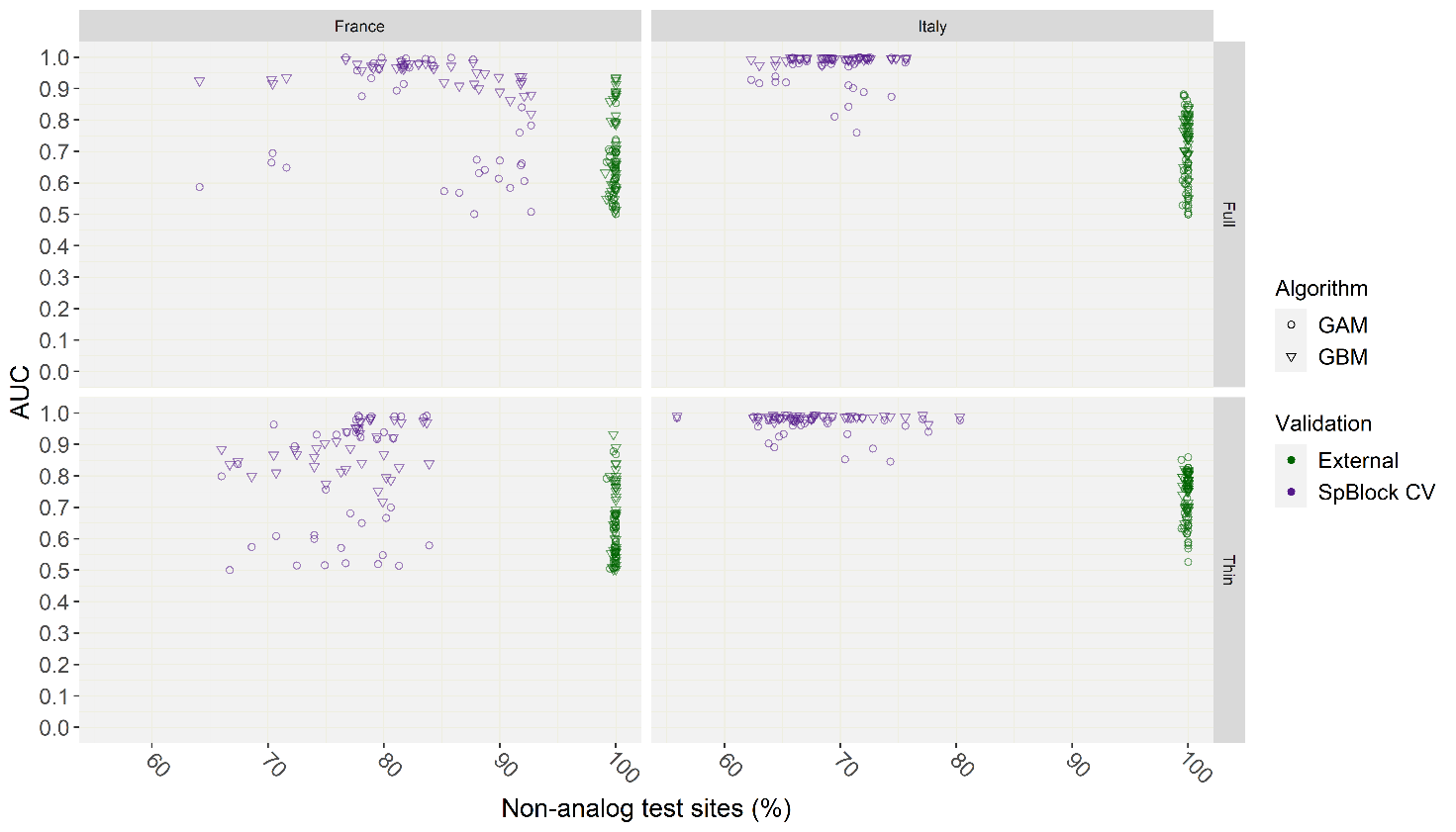


**Figure S4.** (a) Scatterplot showing variation in Continuous Boyce Index scores attained by the HSMs fitted through the GAM (circles) and GBM (triangles) algorithms, using the ‘Ecol’ or ‘VIF’ set of predictors, at increasing level of multivariate novelty in test data, within ‘SpBlock CV’ (purple) and ‘External validation’ (green). (b) Scatterplot showing variation in Continuous Boyce Index scores attained by the HSMs fitted through the GAM (circles) and GBM (triangles) algorithms, using the ‘Full’ or ‘’Thin’ occurrence datasets, at increasing level of multivariate novelty in test data, within ‘SpBlock CV’ (purple) and ‘External validation’ (green).


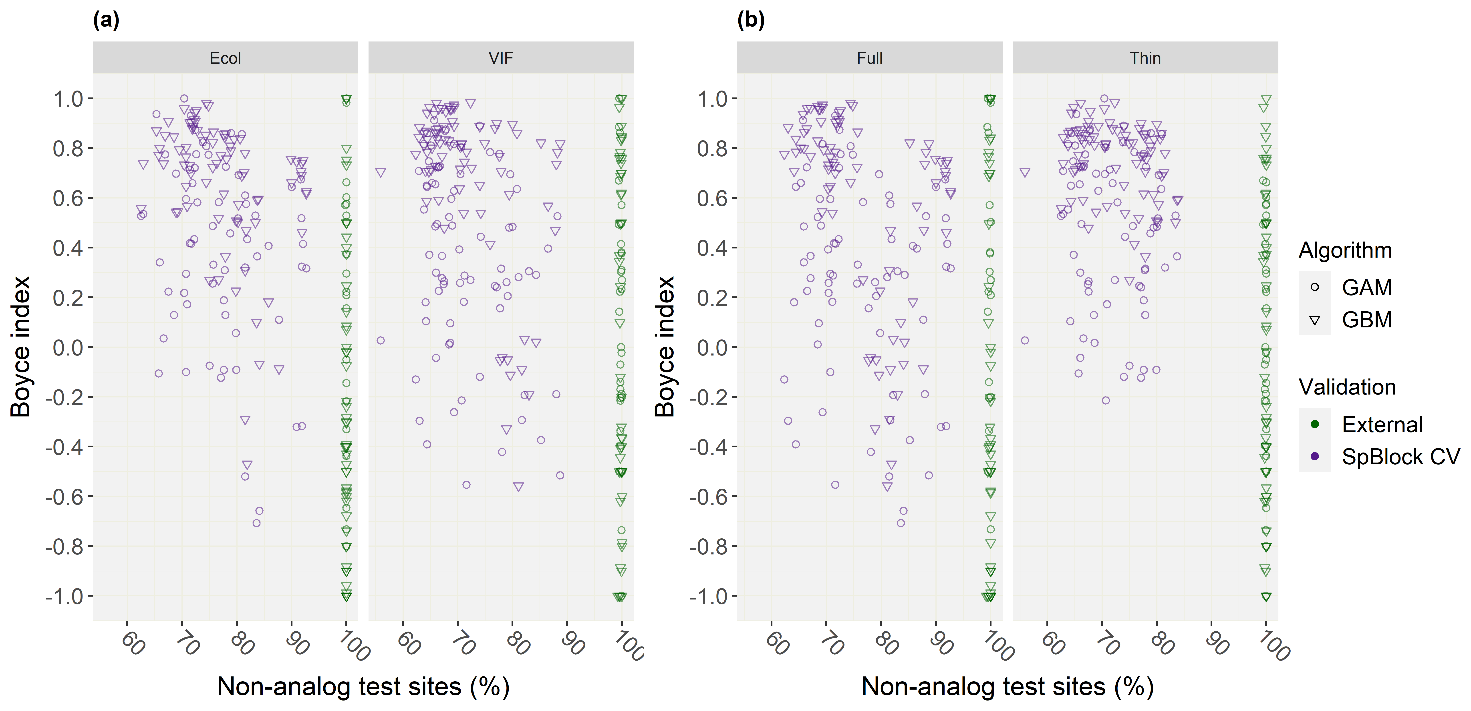


**Figure S5.** Ensemble projection of weighted average suitability derived from the HSMs fitted upon the full occurrence datasets, for each combination of calibration group (‘France’, ‘Italy’, ‘Joint’) * set of predictors (‘Ecol’, ‘VIF’), and obtaining Continuous Boyce Index ≥ 0.7 when validated on spatially independent test samples drawn from the calibration region (‘SpBlock CV’): (a1) ’France-Ecol’, (a2)’France-VIF’, (b1) ’Italy-Ecol’, (b2) ’Italy-VIF’, (c1) ‘Joint-Ecol’, (c2) ‘Joint-VIF’.


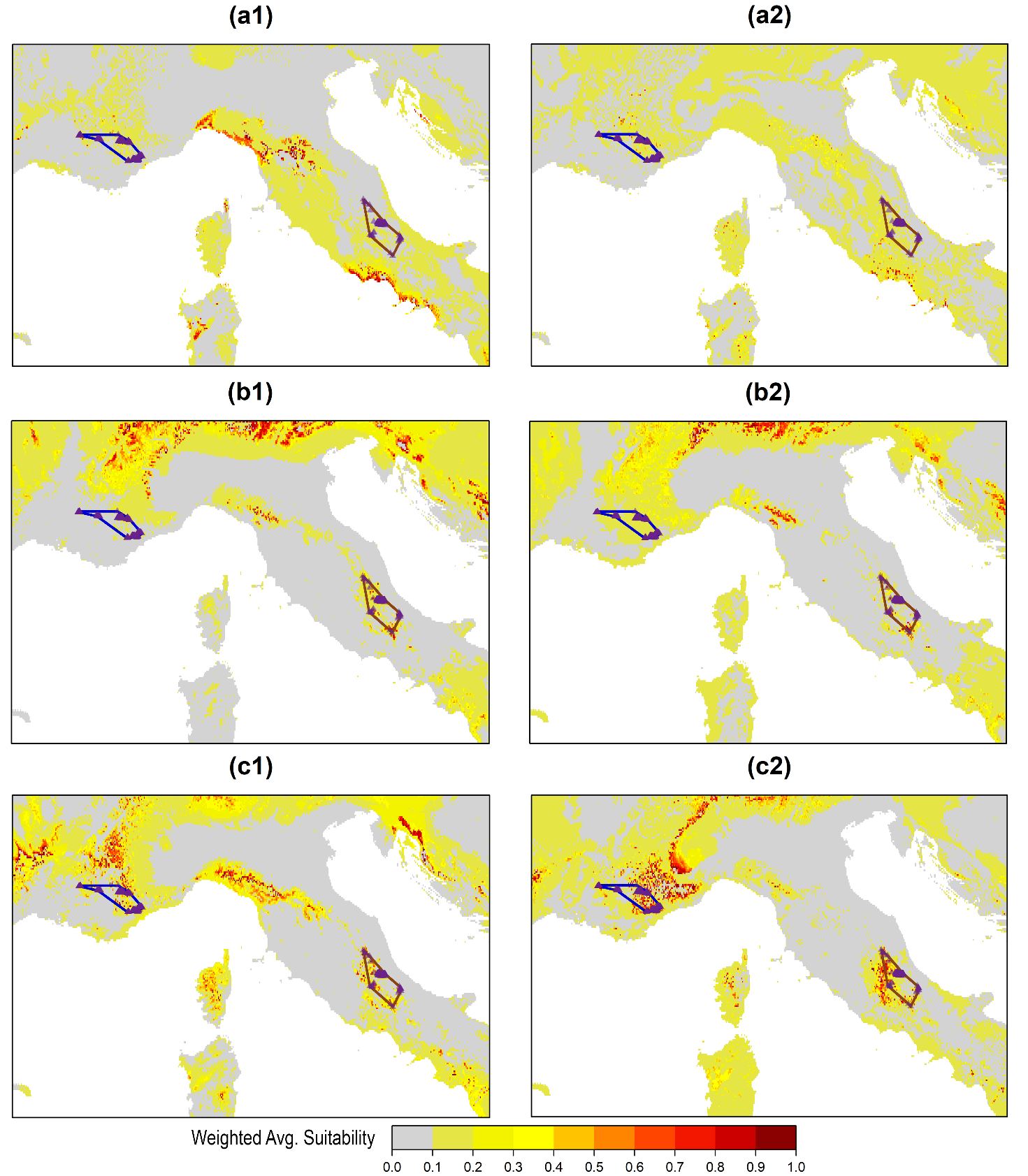


**Figure S6.** Weighted standard deviation of the suitability values predicted by the single HSMs fitted upon the fulll occurrence datasets, for each combination of calibration group (‘France’, ‘Italy’, ‘Joint’) * set of predictors (‘Ecol’, ‘VIF’), and obtaining Continuous Boyce Index ≥ 0.7 when validated on spatially independent test samples drawn from the calibration region (‘SpBlock CV’): (a1) ’France-Ecol’, (a2)’France-VIF’, (b1) ’Italy-Ecol’, (b2) ’Italy-VIF’, (c1) ‘Joint-Ecol’, (c2) ‘Joint-VIF’.


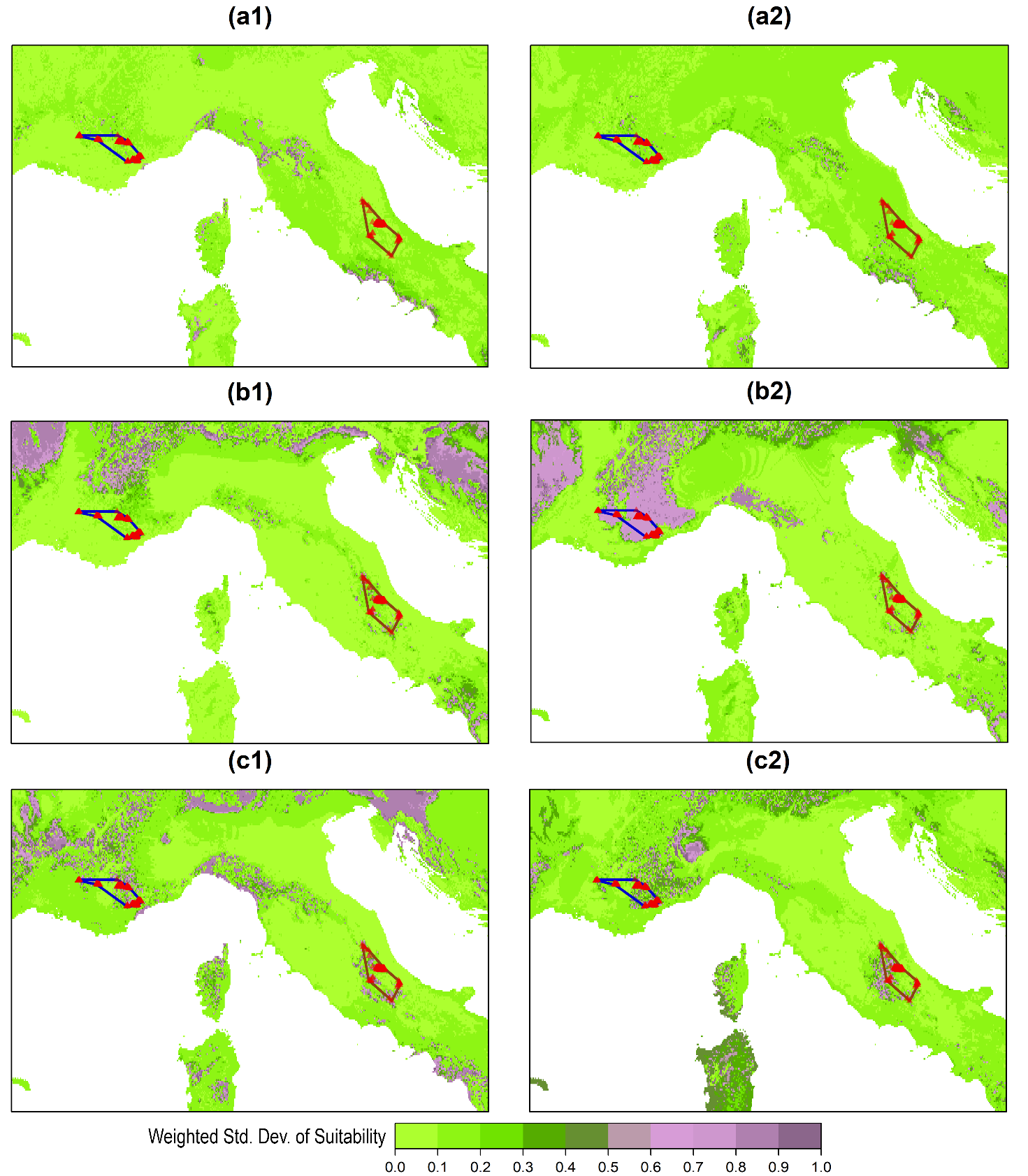


**Figure S7.** Inflated response curves of the predictors showing the three lowest coefficients of variation of importance score among the ones attaining the five highest weighted average importance scores (see Table 2). Curves are extracted from the HSM obtaining the highest Continuous Boyce Index score in ‘SpBlock CV’ among the ones fitted upon the ‘France’ calibration group, for each combination of occurrence data size (‘Full’, ‘Thin’) * set of predictors (‘Ecol’, ‘VIF’): (a) ‘Full-Ecol’; (b) ‘Full-VIF’; (c) ‘Thin-Ecol’; (d) ‘Thin-VIF’.


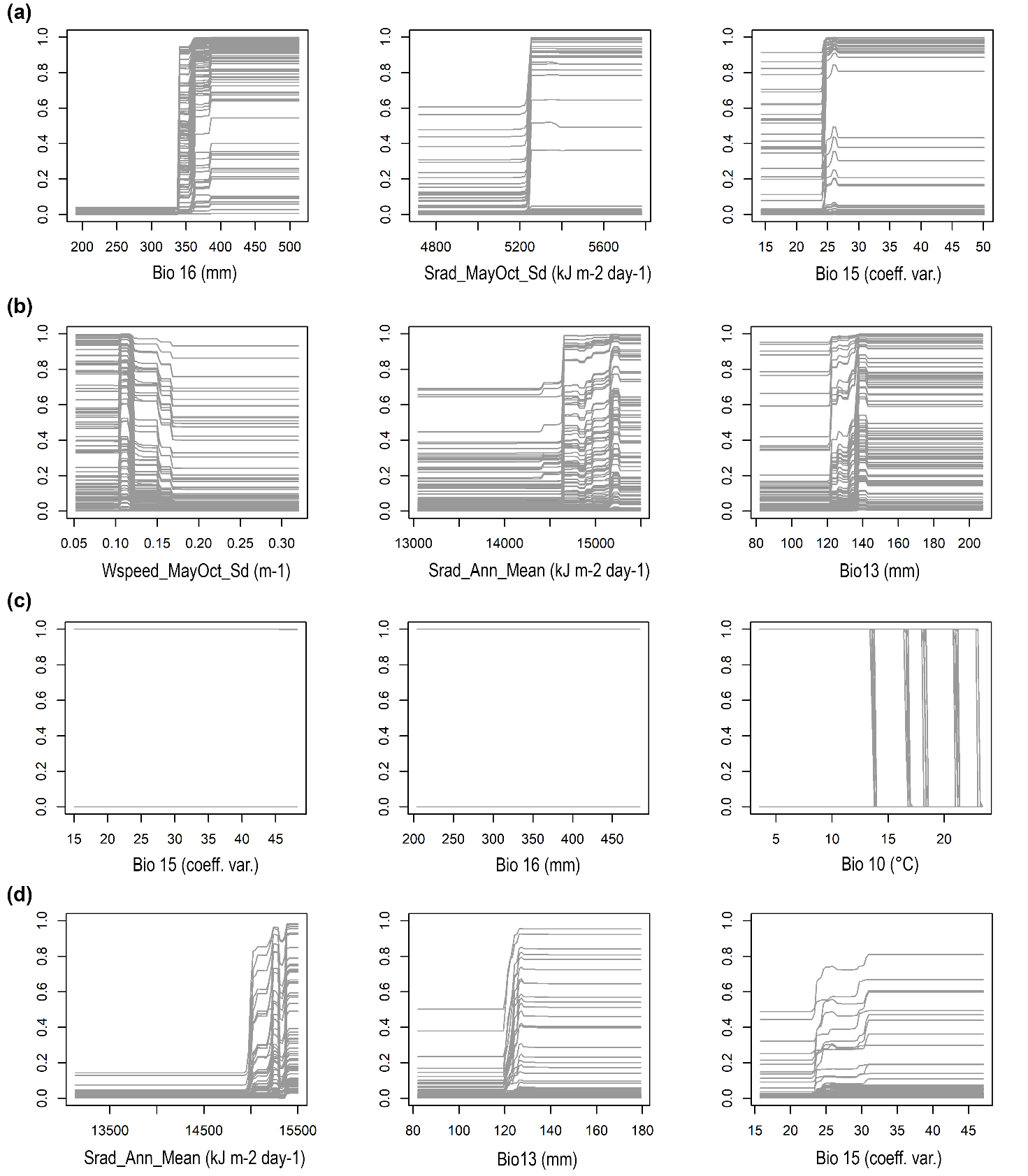


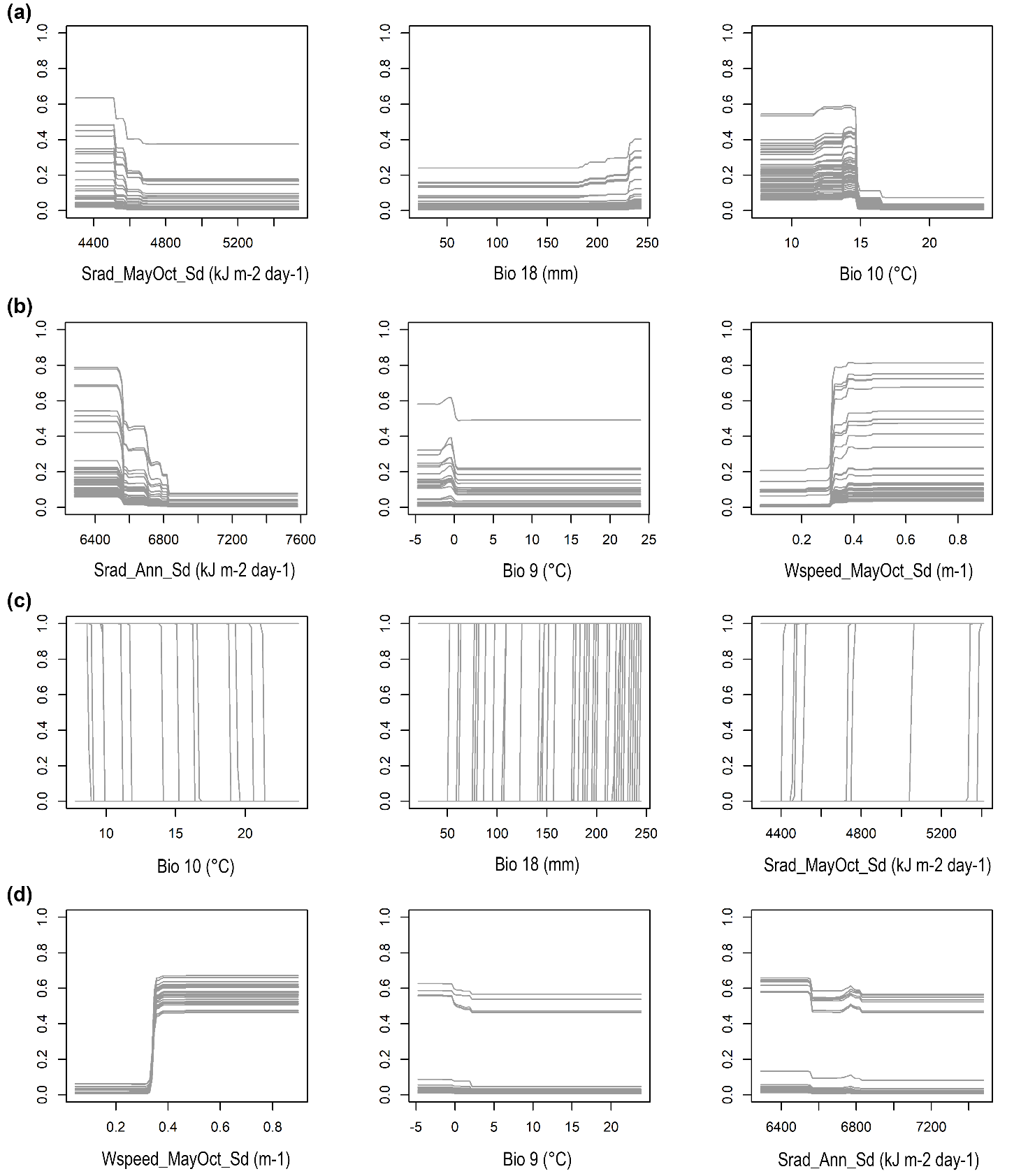
**Figure S8.** Inflated response curves of the predictors showing the three lowest coefficients of variation of importance score among the ones attaining the five highest weighted average importance scores (see Table 2). Curves are extracted from the HSM obtaining the highest Continuous Boyce Index score in ‘SpBlock CV’ among the ones fitted upon the ‘Italy’ calibration group, for each combination of occurrence data size (‘Full’, ‘Thin’) * set of predictors (‘Ecol’, ‘VIF’): (a) ‘Full-Ecol’; (b) ‘Full-VIF’; (c) ‘Thin-Ecol’; (d) ‘Thin-VIF’.

**Figure S9.** Inflated response curves of the predictors showing the three lowest coefficients of variation of importance score among the ones attaining the five highest weighted average importance scores (see Table 2). Curves are extracted from the HSM obtaining the highest Continuous Boyce Index score in ‘SpBlock CV’ among the ones fitted upon the ‘Joint’ calibration group, for each combination of occurrence data size (‘Full’, ‘Thin’) * set of predictors (‘Ecol’, ‘VIF’): (a) ‘Full-Ecol’; (b) ‘Full-VIF’; (c) ‘Thin-Ecol’; (d) ‘Thin-VIF’.


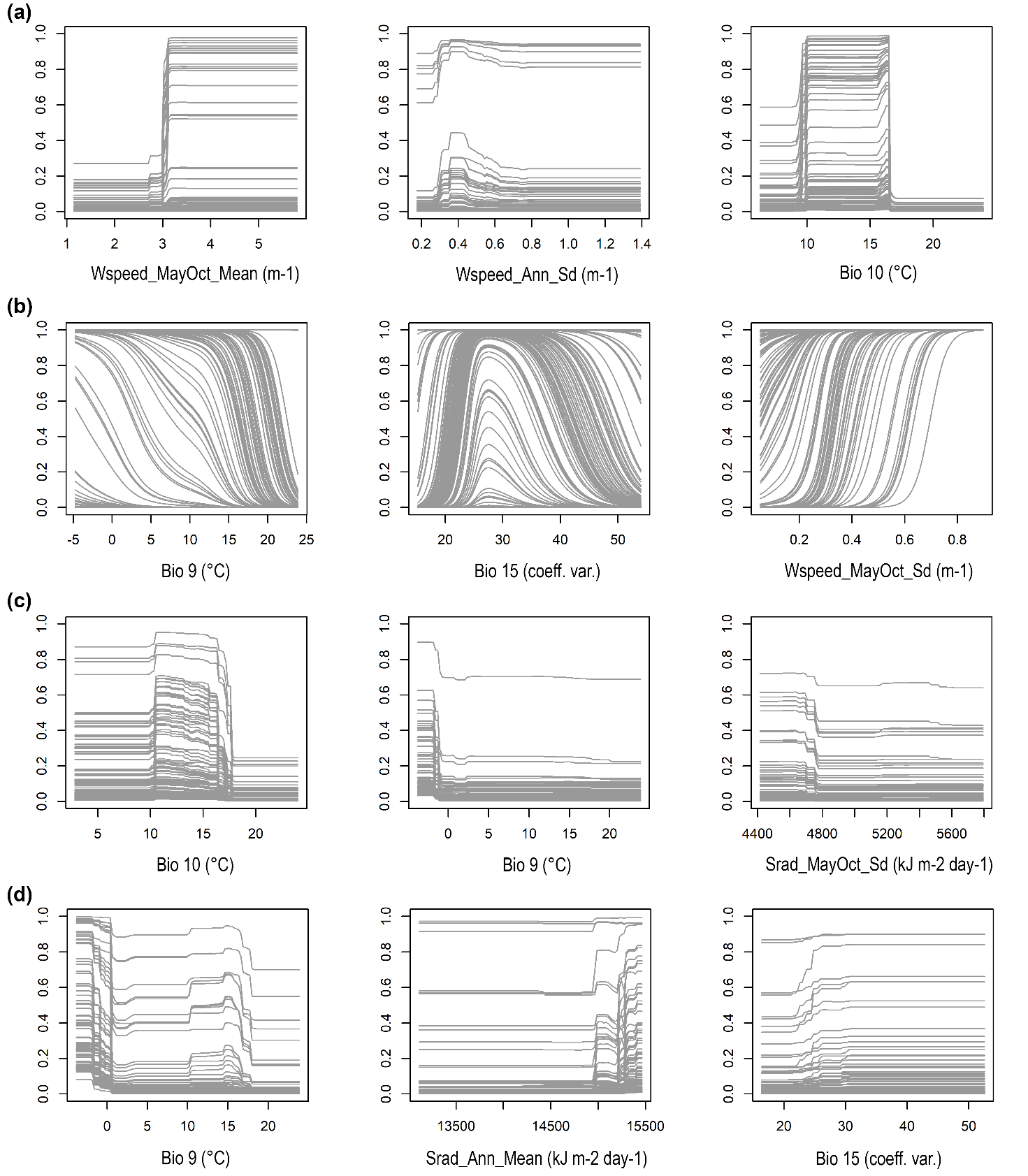

Supplement: Supplementary file 1 — Supplementary Material [file ECE3-11-3991-s002.docx]
